# Analysis of regulatory sequences in exosomal DNA of NANOGP8

**DOI:** 10.1371/journal.pone.0280959

**Published:** 2023-01-25

**Authors:** Manjusha Vaidya, Jonhoi Smith, Melvin Field, Kiminobu Sugaya

**Affiliations:** 1 Burnett School of Biomedical Sciences, College of Medicine, University of Central Florida, Orlando, FL, United States of America; 2 AdventHealth Cancer Institute, Orlando, FL, United States of America; Universitat des Saarlandes, GERMANY

## Abstract

Exosomes participate in intercellular communication by transporting functionally active molecules. Such cargo from the original cells comprising proteins, micro-RNA, mRNA, single-stranded (ssDNA) and double-stranded DNA (dsDNA) molecules pleiotropically transforms the target cells. Although cancer cells secrete exosomes carrying a significant level of DNA capable of modulating oncogene expression in a recipient cell, the regulatory mechanism is unknown. We have previously reported that cancer cells produce exosomes containing NANOGP8 DNA. NANOGP8 is an oncogenic paralog of embryonic stem cell transcription factor NANOG and does not express in cells since it is a pseudogene. However, in this study, we evaluated NANOGP8 expression in glioblastoma multiforme (GBM) tissue from a surgically removed brain tumor of a patient. Significantly higher NANOGP8 transcription was observed in GBM cancer stem cells (CSCs) than in GBM cancer cells or neural stem cells (NSCs), despite identical sequences of NANOGP8-upstream genomic region in all the cell lines. This finding suggests that upstream genomic sequences of NANOGP8 may have environment-dependent promoter activity. We also found that the regulatory sequences upstream of exosomal NANOGP8 GBM DNA contain multiple core promoter elements, transcription factor binding sites, and segments of human viruses known for their oncogenic role. The exosomal sequence of NANOGP8-upstream GBM DNA is different from corresponding genomic sequences in CSCs, cancer cells, and NSCs as well as from the sequences reported by NCBI. These sequence dissimilarities suggest that exosomal NANOGP8 GBM DNA may not be a part of the genomic DNA. Exosomes possibly acquire this DNA from other sources where it is synthesized by an unknown mechanism. The significance of exosome-bestowed regulatory elements in the transcription of promoter-less retrogene such as NANOGP8 remains to be determined.

## Introduction

Glioblastoma multiforme (GBM) is the most aggressive and the least curable of all the malignant tumors of the central nervous system, with a 5-year survival of 6.8% and mean survival of fewer than 18 months [1]. Like many other cancers, GBM possesses a small population of cancer stem cells (CSCs) responsible for cancer recurrence, metastasis, immune evasion, and chemo-radiation resistance [2, 3]. The presence of CSCs is one of the cardinal reasons for GBM’s poor prognosis.

Research suggests that extracellular vesicles play a crucial role in imparting therapy resistance to the CSCs in GBM [4, 5]. Exosomes, the innate extracellular nanovesicles (30–130 nm) originated from late endosomes are secreted by cells possessing an endomembrane system. They can cross the blood-brain barrier and other anatomical compartments via transcytosis [6, 7]. Exosomes participate in paracrine and endocrine intercellular communication by transporting functionally active and pleiotropically effective molecular cargo comprising DNA, RNA, and proteins [8, 9]. CSCs have been shown to produce more exosomes than other cell types and the extent of exosome secretion by the CSCs directly correlates with GBM’s ability to invade adjacent brain regions [10]. As such, anomalies of exosomal DNA released by cancer cells serve as diagnostic markers and play a role in the neoplastic initiation, progression, and metastasis of the disease [11, 12].

NANOG, a homeobox transcription factor, is the crucial regulator of embryonic stemness and pluripotency of mammalian cells [13, 14]. Our previous study showed that NANOG is expressed in GBM CSCs [15]. Further investigation revealed that most of the NANOG expression belonged to NANOGP8, an intronless oncogene generated by retrotransposition of the parent gene NANOG [16–19]. NANOGP8 plays a major role in maintaining CSC within GBM tumors [20]. Although NANOG has eleven paralogs, NANOGP8 is the only one that can encode a full-length protein, differing from the original gene in 2 out of 305 amino acids [21]. In the present study, the restriction fragment length polymorphism (RFLP) analysis of the PCR amplified NANOG cDNA has confirmed the presence of NANOGP8 as well as NANOG transcripts in all the cell samples tested. AlwNI digests NANOGP8 gene, but the parent gene NANOG cannot be digested due to a single nucleotide polymorphism at the cutting site [22–24]. The transcription of NANOGP8 is intriguing since retrogenes typically lack a functional promoter or a regulatory region [25–27]. The National Center for Biotechnology Information (NCBI) does not report annotated promoter region for NANOGP8.

Previous work from our lab has identified differential DNA sequences of NANOG/NANOGP8 exons and 3’ UTRs in GBM exosomes [22]. The molecular cargo of cancer cell-secreted exosomes, including DNA, influences oncogene expression in the recipient cells, indicating that exosomal DNAs may have with promotor [28, 29]. As avobe mentioned, CSCs express intronless oncogene NANOGP8. For a transcriptionally viable gene, the corresponding genomic region possesses regulatory elements. Therefore, we hypothesize that exosomal NANOGP8 DNA secreted by CSCs may have promotor like sequence in its upstream.

In the current study, we analyzed the NANOGP8 DNA 5’ UTR in CSC-secreted exosomes for the presence of core promoter elements and the transcription factor binding sites (TFBS) and compared with the genomic counterpart. Viruses, such as human papillomavirus (HPVs), human cytomegalovirus (HCMV), and neurotropic Epstein-Barr virus (EBV), are implicated in oncogenesis [30, 31]. HPV presence in GBM has a prognostic value [32]. HCMV is reported to induce human retrovirus transcription, which contributes to tumor development [33]. Since viral sequences potentially confer transcriptional role to regulatory regions in a nuclear genome, we also analyzed upstream sequences of exosomal NANOGP8 DNA for the viral sequence inundations.

## Materials and methods

**Fig 1:**
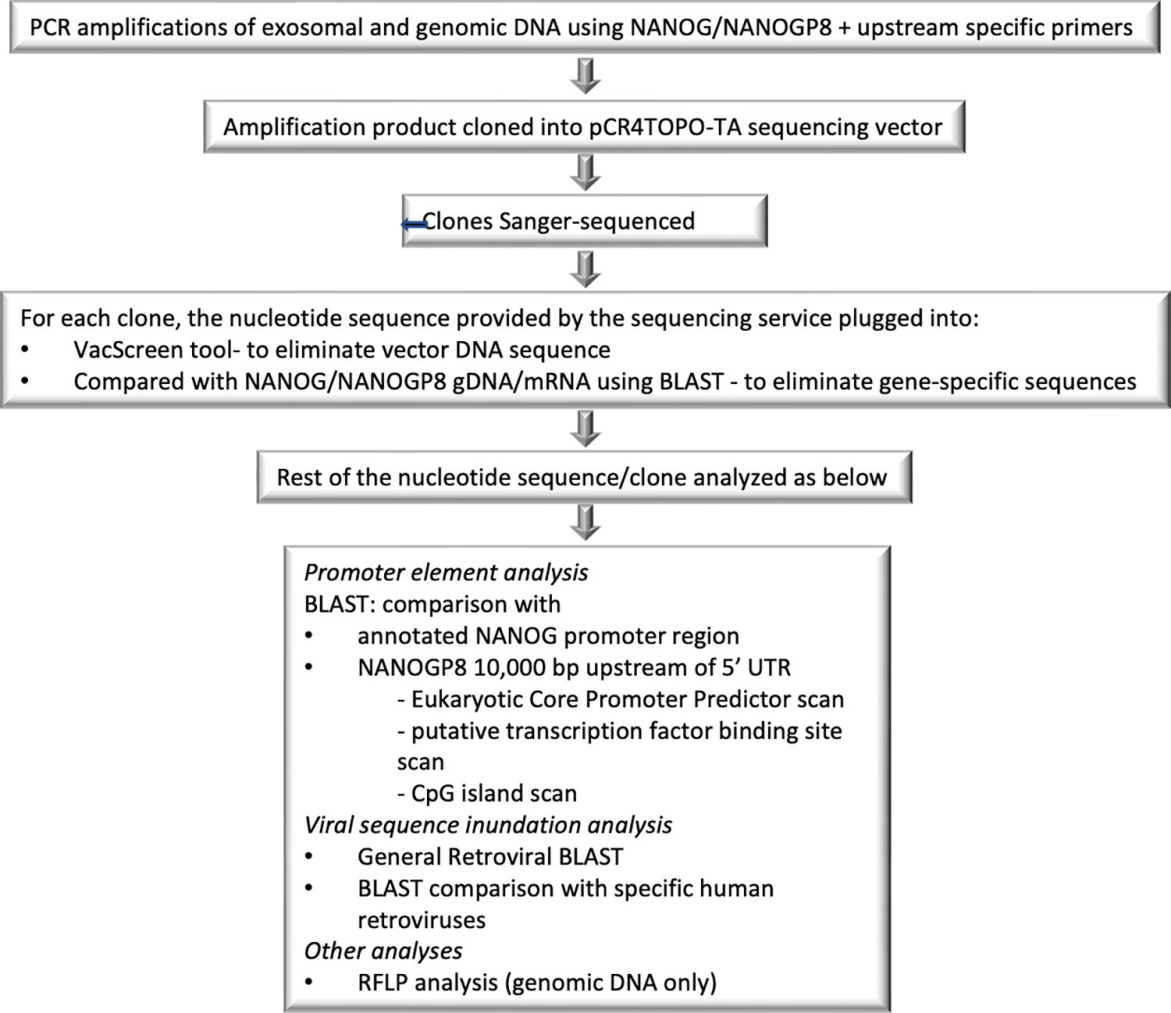
Schematic of the steps in exosomal and genomic DNA sequence analysis.

The exosomal and the genomic DNA was PCR amplified and the PCR products were cloned in a sequencing vector. The clones selected on Ampicillin were Sanger-sequenced and the sequences were analyzed using the Basic Local Alignment Search Tool (BLAST: https://blast.ncbi.nlm.nih.gov/Blast.cgi). A concise account of the steps followed in the NANOGP8 DNA sequence analysis is given in **Fig 1**. The transcription levels of NANOGP8 were checked with quantitative PCR of the cDNA template. To confirm the identities of NANOGP8, RFLP analysis was conducted on the PCR products of cDNA as well as gDNA. The nucleotide sequence analysis (GENEWIZ®) of each clone was plugged into NCBI’s VacScreen tool to eliminate the pCR4TOPO-TA vector sequence contamination that would skew the results of the analysis (https://www.ncbi.nlm.nih.gov/tools/vecscreen/). To remove the possible NANOG/NANOGP8 intron, exon, and UTR sequences from the promoter element analysis, the non-vector exosomal DNA sequence of each clone was compared with NANOG and NANOGP8 using NCBI’s Basic Local Alignment Tool (BLAST). The details of NCBI reference numbers used in comparative BLAST analyses are given in **Table 1**.

**Table 1:** NCBI GENBANK reference numbers for NANOGP8 and NANOG sequences used during BLAST analysis.

	NCBI reference numbers used in BLAST analysis
NANOGP8	NANOGP8 –Homo sapiens chromosome 15, GRCh38.p13 Primary Assembly (NC_000015.10)
	Homo sapiens Nanog homeobox retrogene P8 (NANOGP8) mRNA (NM_001355281.2)
	NANOGP8 Promoter region (1000nt) + 5’UTR + exon+3’UTR (35082410 to 35085408)
NANOG	NANOG–Homo sapiens chromosome 12, GRCh38.p13 Primary Assembly (NC_000012.12)
	NANOG mRNA Homo sapiens Nanog homeobox (NANOG), transcript variant 1 (NM_024865.4)
	NANOG promoter region: Homo sapiens chromosome 12, GRCh38.p13 Primary Assembly [NC_000012.12 (7789402 to 7799146)]

### Cell culture

Human NSCs from Lonza (# PT2599) were propagated in suspension cultures, in NSC media (recipe below). GBM cells were isolated from a freshly resected tumor sample of a patient with pathology-confirmed GBM. The tumor tissue was acquired under an AdventHealth and University of Central Florida Institutional Review Board-approved human subject research protocol. Written informed consent was signed by the patient. We strictly followed HIPPA regulations. GBM cells were dissociated from the tumor for in vitro culture. The connecting tissue and blood were removed from the tumor tissue. The tumor tissue was further chopped into finer pieces (less than 1mm) in a petri dish kept cold on ice. The chopped pieces were incubated with Trypsin-EDTA for 10 min at 37°C with 5% CO_2_ and the reaction was stopped with serum containing NT2 media (recipe below). The cells were then passed through a cell strainer using NT2 media to facilitate cell passage. Collected flow through was centrifuge at 4°C, at 4000*g* for 5 minutes, and the cell pellet was washed with NT2 media. After 2 washes, the cell pellet was finally resuspended in 10 ml of NT2 media and transferred to tissue culture treated T-75 flask for overnight incubation at 37°C with 5% CO_2_. The following day, floating spheres of cells were transferred to a non-treated (suspension culture) T-75 flask containing NSC media. After 2 weeks of incubation, the adherent cells were trypsinized and passaged for propagation. The GBM neurospheres in suspension culture, containing a mixed population of cancer cells and CSCs, were subjected to immuno-separation using CD133, a stemness marker, to collect CSCs [15]. CD133^+^ CSCs were separated from the hetero-cellular GBM neurospheres using CD133 conjugated magnetic beads (Miltenyi Biotec, CD133 microbeads, human, Catalog No. 120-000-312), following the manufacturer’s protocol. GBM CDCs were further propagated in suspension cultures. The growth media for GBM and NSC suspension cultures consisted of Heparin 5000 U (0.5 U / mL), EGF 20 ng/mL, bFGF 20 ng /mL, and 2% B27 stock mixed in Dulbecco’s Modified Eagle Medium/Nutrient Mixture F-12 (DMEM-F12). To propagate mixed GBM cells, we cultured them under adherent conditions using NT2 media comprising DMEM-F12 supplemented with 10% exosome-depleted fetal bovine serum (FBS). All the media were supplemented with penicillin and streptomycin (100 U / mL of each). The conditioned media was then collected for exosomal isolation.

### Exosome isolation

Cell culture-conditioned media was centrifuged at 10000xg for 30 minutes to remove cell debris. 10 ml of supernatant was then transferred to a new 50 ml tube with 5 ml of 20% PEG and 200 μL of 7.5 mM NaCl and incubated overnight at 4˚C [34]. The next day, the supernatant was centrifuged at 10000xg for 60 minutes at 4˚C to form an exosome pellet. The pellet was re-suspended in PBS (pH 7.4, without Calcium and Magnesium) and further purified using CD63 antibody-conjugated magnetic beads (Thermo Fisher Scientific, #10606D) using the manufacturer’s protocol. Immunoaffinity-based purification using tetraspanin family member CD63 was used to confirm the presence of exosomes as opposed to any other extracellular vesicle [35–37].

### PCR amplification of exosomal DNA

As previously described, exosomes attached to the CD63-coated magnetic beads were used directly as a template for PCR without isolation of exosomal DNA [22]. Universal forward primer (5’-CACTATGCTCATA-3’) and the reverse primer (5’- GTAGGTAGGGTGCTGAGGC-3’) at position 690–707 on NANOGP8 mRNA (NM_001355281.2), were employed for amplification. Using High-Performance GoTaq® G2 DNA Polymerase (Promega), the PCR reactions were set up as follows: 94˚C -5 minutes, (denaturation: 94˚C-30 seconds, annealing: 48˚C-30 seconds, Extension: 72˚C- 2 minutes) x 30 cycles, 72˚C -10 minutes. The PCR products were electrophoresed on 1.5% TEA-agarose gel. The DNA was eluted from the gel with a QIAquick PCR purification Kit (Qiagen, Catalog #/ID: 28104), using the manufacturer’s protocol. The purified PCR product was reamplified with the same primers and conditions except at higher annealing temperature: 94˚C -5 minutes, (denaturation: 94˚C-30 seconds, annealing: 55˚C-30 seconds, Extension: 72˚C-2 minutes) x 30 cycles, 72˚C -10 minutes. The resulting PCR products were cloned into a vector for sequence analysis.

### Genomic DNA—isolation

Genomic DNA of NSCs and CD133^+^ GBM CSCs was extracted and purified using QIAamp® genomic DNA kit (Qiagen, Catalog # 51304) following the manufacturer’s protocol. The concentration of gDNA was measured with the NanoDrop 8000 spectrophotometer (Thermo Scientific, Waltham, MA, USA) at 260nm.

### PCR amplification of genomic DNA

25 ng of genomic DNA (gDNA) was amplified using the forward primer (NANOGP8-F: 5’-GGCGTCAGGTAGATGGCAAG-3’) and reverse primer (NANOGP8 -R: 5’-CAACCAGCTCAGTCCAGCAGAA-3’). The primer locations of the forward and the reverse primers are 35085783 (promoter region of NANOGP8) and 35085802 (NANOGP8) on NC_000015.10, Homo sapiens chromosome 15, GRCh38.p13, respectively. The NANOGP8-specific reverse primer is located in 5’ UTR and the forward primer was designed with a sequence from the upstream region of the gene reported in the NCBI database (NCBI Reference Sequence: NC_000015.10: 35085802–35085783). The amplified sequences were analyzed using Human BLAST. Using High-Performance GoTaq® G2 DNA Polymerase (Promega), the PCR reactions were set up as follows: 94˚C -5 minutes, (denaturation: 94˚C-30 seconds, annealing: 56˚C-30 seconds, Extension: 72˚C- 30 seconds) x 35 cycles, 72˚C—5 minutes.

### RFLP analysis of gDNA PCR fragments to confirm NANOGP8 and the upstream region

To confirm that the PCR product belongs to the genomic DNA of NANOGP8 and its upstream region, we performed an RFLP analysis using RE HpyF3I. This enzyme recognizes the nucleotide sequence 5’-C^V^TNAG and cut the PCR product in two places yielding three fragments of sizes 234, 287, and 8 bp. We digested 10μL of unpurified gDNA PCR product by endonucleases HpyF31 (Thermo Scientific™ Catalog number: FD1884) for 10 min at 37˚C. The digestion reactions were immediately electrophoresed on a 2% agarose gel in TEA buffer.

### Cloning of PCR products in pCR4TOPO-TA vector

Amplified PCR products from gDNA and exosomal DNA were cloned and sequenced using the same protocol. After gel electrophoresis, the DNA samples were eluted using QIAquick® Gel Extraction Kit (Qiagen catalog # 28706). PCR products were ligated at room temperature with pCR4TOPO-TA vector (Invitrogen™ TOPO™ TA Cloning™ Kit for Sequencing, catalog # 450030), using the manufacturer’s protocol. Chemically competent E. coli (Stbl3) cells were transformed with ligations using the heat-shock method, plated on LB agar with 100 μg/mL ampicillin, and incubated at 37˚ overnight. The bacterial colonies were picked and grown in LB with 100 μg/mL ampicillin. Each colony represents a single clone. The plasmid DNA was extracted from the bacterial pellet using QIAprep Spin Miniprep Kit (QIAGEN #27104). Using M13 reverse primer, clones were Sanger sequenced (GENEWIZ®). The glycerol stocks of bacterial clones were stored at -80˚C.

### Quantitative PCR amplification of NANOGP8 transcript

Total RNA was extracted from all three types of GBM cells as well as NSCs using the Direct-zol RNA extraction kit (Zymo Research, catalog # R2050), following the manufacturer’s protocol. 2.5μg of RNA was synthesized to cDNA with oligo(dt), using the SuperScript™ III First-Strand Synthesis System (Invitrogen) Fisher scientific catalog # 18080051). RNA was incubated with Oligo(dt) at 65 ˚C for 5min, followed by an RNA conversion reaction that proceeded as follows: 25˚C for 10 min, 50˚C for 50 min, and 85˚C for 5 min. For qPCR, 125 ng of cDNA was amplified in 20 μL reactions using Fast SYBR Green Master Mix (Applied Biosystems, ThermoFisher catalog # 4385612). NANOGP8 expression was measured using NANOGP8 specific primers (qNANOGP8 F: 5’-TTTGTGGGCCTGAAGAAAACT-3’, qNANOGP8R: 5’-AGGGCTGTCCTGAATAAGCAG-3’). B-actin-specific primers (qB-Actin F: 5’-AGAGCTACGAGCTGCCTGAC-3’, qB-Actin R: 5’-AGCACTGTGTTGGCGTACAG) amplified the gene that served as the endogenous control. qPCR reaction was performed in the QuantStudio™ 7 Flex Real-Time PCR System (Applied Biosystems). Amplification was quantified by SYBR green fluorescence and normalized based on the ROX passive reference dye. Thermocycling program for the amplification reaction is: 95˚C- 20 sec [95˚C -1 sec, 62˚C—20 sec] x 40 cycles. We measured relative NANOGP8 expressions using the 2^-ΔΔCT^ Livak-method and performed statistical analysis one-way ANOVA followed by post-hoc Dunnett’s test employing GraphPad Prism (v9.3.0) statistical program.

### PCR amplification of NANOG transcript

We amplified the total cDNA of the cell samples using NANOG/NANOGP8 primers (Forward: 5’-GTCTTCTGCTGAGATGCCTCACA-3’ and Reverse: 5’-CTTCTGCGTCACACCATTGCTAT-3’ Position on NANOGP8 gene- GenBank Accession: 388112: 418–802). Using High-Performance GoTaq® G2 DNA Polymerase (Promega), the PCR reactions were set up as follows: Pre-denaturation: 94˚C -7 minutes, (denaturation: 94˚C-30 seconds, annealing: 55˚C-30 seconds, Extension: 72˚C-2 minutes) x 35 cycles, post extension: 72˚C -10 minutes). The amplified PCR product was digested with AlwNI at 37˚C for 5 minutes and run on 2% TEA-agarose gel.

### RFLP analysis to confirm NANOG/NANOGP8 transcript

The DNA of the PCR product was extracted from the gel using QIAquick® Gel Extraction Kit (Qiagen catalog # 28706) following the manufacturer’s protocol. 1 μg of amplicon DNA was digested with restriction enzymes AlwNI (also known as CaiI, ThermoFisher, Fast Digest enzyme, catalog # FERER1391) with Fast Digest buffer. The digestion reaction was incubated at 37° C for five minutes and electrophoresed on 3% Agarose gel in 1X TAE buffer.

### Core promoter element analysis of exosomal and genomic DNA

Using the NCBI information given in **Table 1**, we confirmed the presence of NANOG DNA in the exosomal clones as well as genomic DNA clones. Upon confirmation of the NANOG or NANOGP8 gene sequence, the upstream sequences were compared with NCBI-reported sequence data. This was done to check their identity to the reported promoter region of NANOG and the upstream region of the NANOGP8 gene. For comparison of exosomal and genomic upstream/regulatory regions, 1000 nucleotides upstream of the 5’UTR were added to the BLAST. Because NANOG possesses an annotated promoter, the NANOGP8 upstream sequences were compared with the promoter region’s genomic sequence. None of the upstream sequences matched with the annotated promoter region of NANOG or NCBI reported 5’ upstream region of NANOGP8.

For the core promoter element analysis, the upstream exosomal NANOG DNA sequences were plugged into YAPP Eukaryotic Core Promoter Predictor (http://www.bioinformatics.org/yapp/cgi-bin/yapp.cgi). This tool scanned the nucleotide sequences for canonical promoter elements and detected the TATA box, to identify transcription-start-site (TSS), downstream promoter element (DPE), and an initiator element (INR). YAPP also identified synergistic combinations of the core promoter elements found in upstream sequences. YAPP algorithm calculates matrix similarity score for matches with consensus sequences to qualify as promoter elements. If each nucleotide in the sequence to be analyzed corresponds to the highest conserved nucleotide in the same position of the matrix, this perfect match receives a score of 1.0. Matrix similarity score >0.8 is considered good and, therefore, the optimum cutoff value in the analysis [38–40]. As reported in Tables 2–4, all the promoter element motifs in our analyses were between 0.8 and 1.

**Table 2:** Summary of TFs for which the binding sites were found in the exosomal DNA.

		Transcription factors for which the binding sites are found in exosomal and genomic DNA clones									
TF binding sites in exosomal DNA **unique** to a cell type	**NSC**	MBF1	EFII	STAT5A	IRF-1	YY1	STAT4	NF-AT2	Elk-1	IRF-3	PEA3
		p300	NF-AT1	c-Ets-2	NF-AT1	R2	NF-AT4	HNF-3beta	Pu box binding factor	NF-AT3	
	**GBM**	MEF-2A									
	**CD133**^**-**^ **GBM**	AP-1	GABP	GABP-alpha							
TF binding sites in exosomal DNA **common** among cell types	**NSC and GBM**	YAP1									
	**NSC and CD133**^**-**^ **GBM**	POU2F1									
	**GBM and CD133**^**-**^ **GBM**	Sp1	RXR-alpha	CRF	MBP-1 (1)	NF-1	NF-kappaB	TEF-1	GR		
		PR B	AP-4	PR-beta	PR-alpha	LVc	LVb-binding factor	c-Myb			
	**Common to all the three cell types**	C/EBPalpha	HMG I(Y)	c-Jun	c-Ets-1	E2F-1	FOXN2	c-Fos	T3R-alpha		

**Table 3:** Viral identities found in the promoter region amplified product of NSC and CD133^+^ GBM gDNA. Standard BLAST of 493 nucleotide-long “Promoter” region revealed that only 275 nucleotides have identities to retroviral sequences, including HIV-1.

BLAST ® » blastn suite » results for RID-T81HY083013		
Description	Scientific Name	Accession
Human endogenous retrovirus type K (HERV-K), gag, pol, and env genes	Human endogenous retrovirus K	Y18890.1
Human Endogenous Retrovirus IDDMK1,2–22 3’ retroviral regulatory terminal end	Human Endogenous Retrovirus IDDMK1,2–22	AF012335.1
Human endogenous retrovirus K113 LTR, complete sequence	Human endogenous retrovirus K113	JF742069.1
Human endogenous retrovirus K115 complete genome	Human endogenous retrovirus K115	AY037929.1
Human endogenous retrovirus K113 complete genome	Human endogenous retrovirus K113	AY037928.1
Human endogenous retrovirus HERV-K(II) DNA, complete sequence, and flanking region	Human endogenous retrovirus HERV-K(II)	AB047240.1
Human endogenous retrovirus HERV-K(I) DNA, complete sequence, and flanking region	Human endogenous retrovirus HERV-K(I)	AB047209.1
Human endogenous retrovirus K (HERV-K) elements, clone C7	Human endogenous retrovirus K	Y17832.2
Human endogenous retrovirus K (HERV-K) elements, clone SD-C7-34LTR	Human endogenous retrovirus K	Y17834.1
Human endogenous retrovirus K (HERV-K) elements, clone gP23-C19	Human endogenous retrovirus K	Y17833.1
Human endogenous retrovirus env mRNA	Human endogenous retrovirus	X82272.1
Human endogenous retrovirus mRNA for central open reading frame	Human endogenous retrovirus	X82271.1
Human endogenous retrovirus mRNA for hypothetical protein	Human endogenous retrovirus	X72790.1
Human endogenous retrovirus HERV-K, LTR R and U5 region and gag gene	Human endogenous retrovirus K	Y10390.1
Human Endogenous Retrovirus K1,2–4 5’ retroviral regulatory terminal end	Human Endogenous Retrovirus K1,2–4	AF012328.1
Human Endogenous Retrovirus IDDMK1,2–22 5’ retroviral regulatory terminal end	Human Endogenous Retrovirus IDDMK1,2–22	AF012332.1
HIV-1 isolate 258_E_6 from Zimbabwe envelope glycoprotein (env) gene, partial cds	Human immunodeficiency virus 1	HQ708051.1
HIV-1 isolate 258_E_23 from Zimbabwe envelope glycoprotein (env) gene, partial cds	Human immunodeficiency virus 1	HQ708048.1

**Table 4:** Summary of common TFBs found in the upstream of NANOGP8 isolated from the gDNA and exosomal DNA clones.

	Common transcription factors for which the binding sites are found in genomic DNA and exosomal DNA clones							
**TFBS common to NSC gDNA, CD133**^**+**^ **GBM gDNA, and exosomal DNA**	STAT5A	IRF-1	YY1	STAT4	NF-AT2	Elk-1	FOXN2	c-Fos
	p300	NF-AT1	c-Ets-2	HMG I(Y)	R2	NF-AT4	HNF-3beta	NF-AT3
	AP-1	POU2F1	RXR-alpha	NF-1	GR	c-Jun	c-Ets-1	E2F-1

Transcription factors (TFs) fall under two major categories; general and specific. General TFs bind to the core promoter region to assist the docking of RNA polymerase for basal transcription, whereas the specific TFs regulate the transcription of an individual gene. We analyzed the exosomal clones for sequences recognized by the latter type. Using a virtual laboratory app, “PROMO” (version 3.2.0), we scanned the upstream sequences of exosomal NANOGP8 DNA for TFBSs (http://alggen.lsi.upc.es/cgi-bin/promo_v3/promo/promoinit.cgi?dirDB=TF_8.3). This program identified the putative binding sites and the TF proteins that bind to them in DNA sequences. The app uses TFBSs defined by the TRANSFAC® eukaryotic TF database (version 8.3) [41, 42].

## Results and analysis

**Fig 2:**
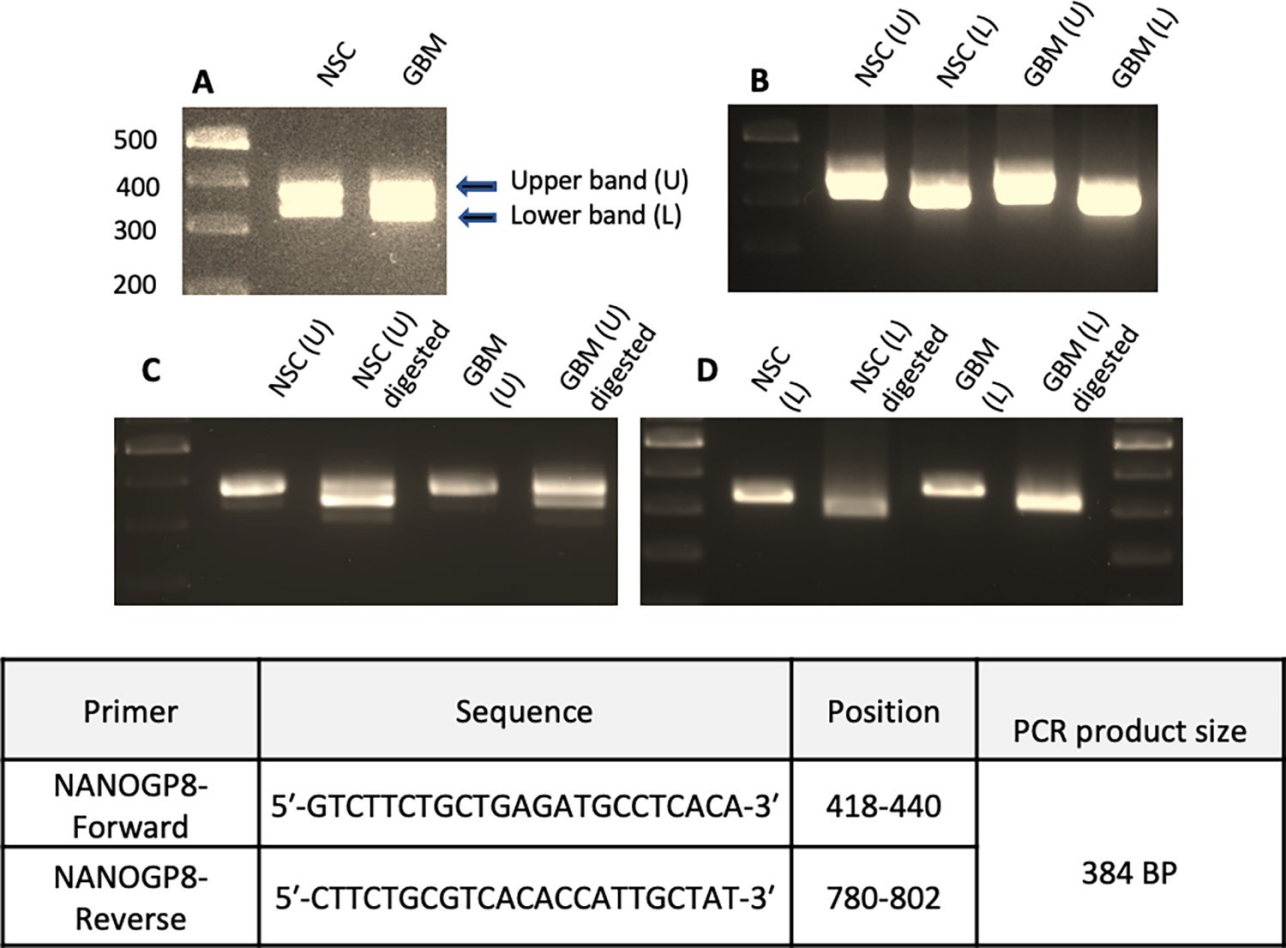
RFLP analysis of NANOGP8 transcript. cDNAs from NSC and GBM were amplified with NANOGP8 primers and digested with RE AlwNI. (**A**) Initial amplification yielding two bands of PCR products, pointed by red arrows are labeled as “U” and “L” for upper and lower bands respectively **(S1 Raw image)**. (**B**) Reamplification of the upper and lower band, separately **(S2 Raw image)**. (**C**) RE-AlwNI digestion of upper bands showing partial digestion, confirming the presence of a mixed population of NANOG and NANOGP8 transcripts **(S3 Raw image)**. **(D)** RE-AlwNI digestion of lower bands showing complete digestion of the PCR product validates that the PCR product belongs to the NANOGP8 transcript alone. Panel D is made by removing the irrelevant lanes from **S4 Raw image**.

The AlwNI-mediated digestion of cDNA PCR products in RFLP analysis confirmed the presence of NANOGP8 transcripts in NSCs and GBM cells. For both the cell lines, the RFLP analysis results were identical. In our samples, the PCR amplification of the transcript using the NANOG/NANOGP8 primer pair unexpectedly yielded two distinct product bands. The upper band was of expected size, i.e. 384 base pairs and the lower band was approximately 340 base pairs. Each band reamplified separately using the same primer pair, followed by a digestion with AlwNI, gave interesting results. The upper band showed partial digestion affirming the presence of a mixed population of NANOG and NANOGP8 transcripts. A complete digestion of the PCR products was observed in the lower band, indicating that the transcript belonged solely to NANOGP8 **[Fig 2]**. Therefore, it is inferred that GBM and NSC have a mixed population of the NANOGP8 transcripts. The difference in the PCR product sizes of the upper and lower band also implies that the distinct populations of NANOGP8 transcript have deletions/additions of about 44 nucleotides.

**Fig 3:**
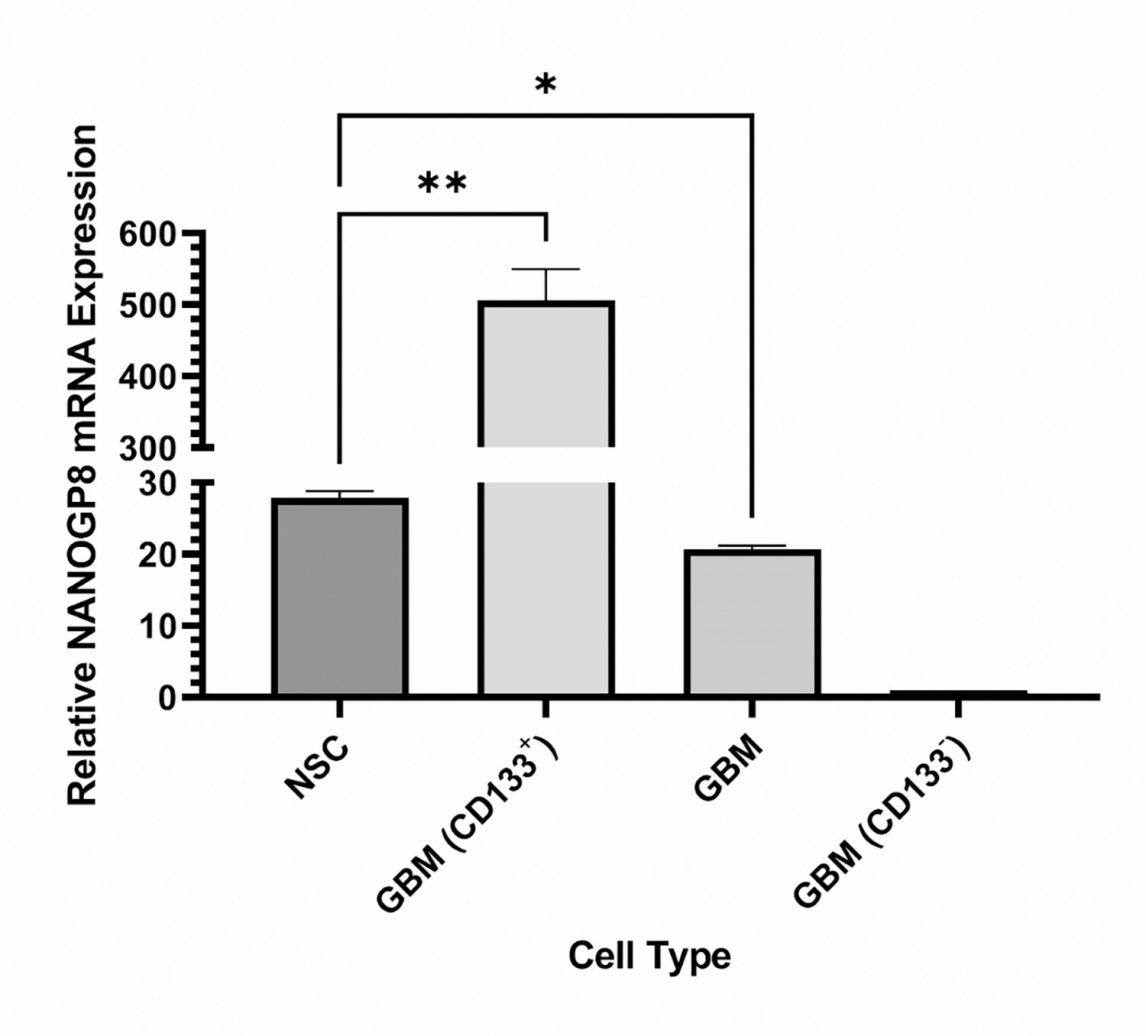
Quantitative PCR amplification of NANOGP8 transcript. CD133^+^ GBM cancer stem cells have significantly higher expression as compared to the GBM primary cells comprising a mix of undifferentiated CD133^+^ CSCs and differentiated CD133^-^ cancer cells. CD133^-^ cells express negligible NANOGP8 transcript.

qPCR mediated transcription analysis demonstrated that CD133^+^ GBM CSCs expressed NANOGP8 approximately 20 times more as compared to NSCs, while CD133^-^ GBM cancer cells expressed only a negligible level of NANOGP8 **[Fig 3]**. We also found that NANOGP8 expression in NSC was remarkably lower (P-value = 0.0056) than the expression in CD133^+^ GBM CSCs, but significantly higher (P-value = 0.0157) than CD133^-^ GBM cells.

Upon detection of upregulated NANOGP8 transcription in CD133^+^ GBM CSCs, we investigated the genomic upstream region of NANOGP8 to check for differential sequences that may have played a role in promoter-like activity in GBM and NSC cell lines. The amplified sequences were analyzed using Human BLAST. The genomic sequences from both the cell lines matched 100% with each other **(S1 Fig)** and showed 99% identity with the reported sequences of the NANOGP8 gene (NC_000015.10), including its upstream region **(S2A and S2B Fig)**.

**Fig 4:**
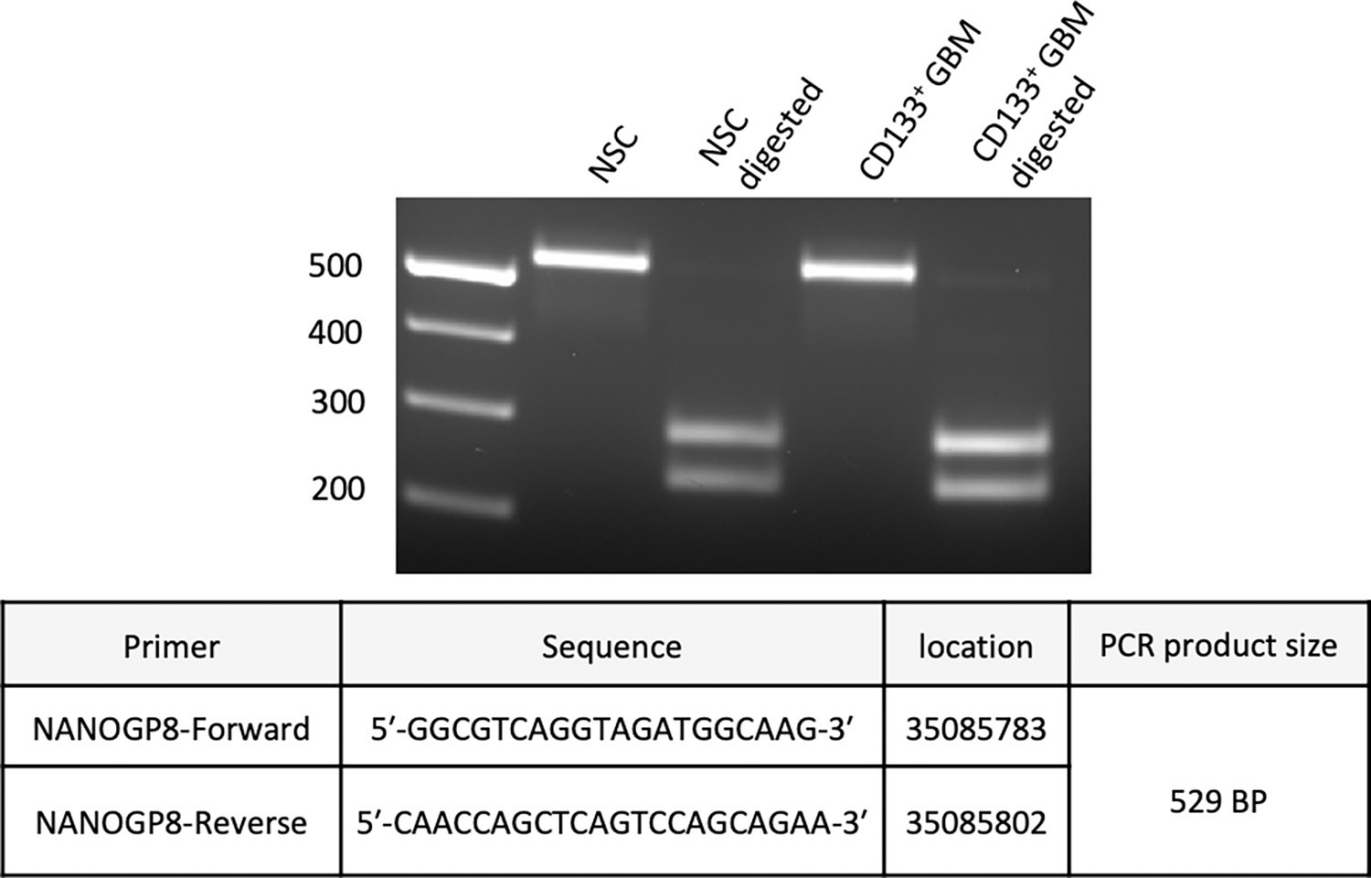
RFLP analysis of NANOGP8 gDNA PCR product. gDNA amplified with NANOGP8 primers. PCR product digested with HpyF3I. NSC and GBM show complete digestion, affirming the presence of the NANOGP8 transcript. A PCR product of 529 bp is digested to yield fragments of 287, 234, and 8 bp each. The 8 bp fragment is not visible in the image. **S5 Raw image** shows the details.

Because the restriction enzyme cutting sites for HpyF31 are present uniquely in the sequence of 5’ UTR and upstream regions of genomic DNA of NANOGP8 (positions 35085287 and 35085520), but not the parent gene NANOG or its other paralogues, a complete digestion with the RE confirmed that the amplification product belonged solely to NANOGP8 **[Fig 4]**.

To analyze the exosomal NAOGP8 DNA and the adjoining upstream sequences, a pair of universal-forward primer and NANOGP8-reverse primer (position on mRNA NM_001355281.2: 690–707) was used on all the exosome samples. Bioinformatical analyses revealed that for both NSC and GBM, NANOGP8- flanking sequences had identities to multiple core promoter motifs, putative transcription factor binding sites (TFBS), and viral elements known to play a role in cancer.

### Core promoter element analysis

#### Analysis of the upstream sequence of NANOG DNA associated with NSC exosomes

**Fig 5:**
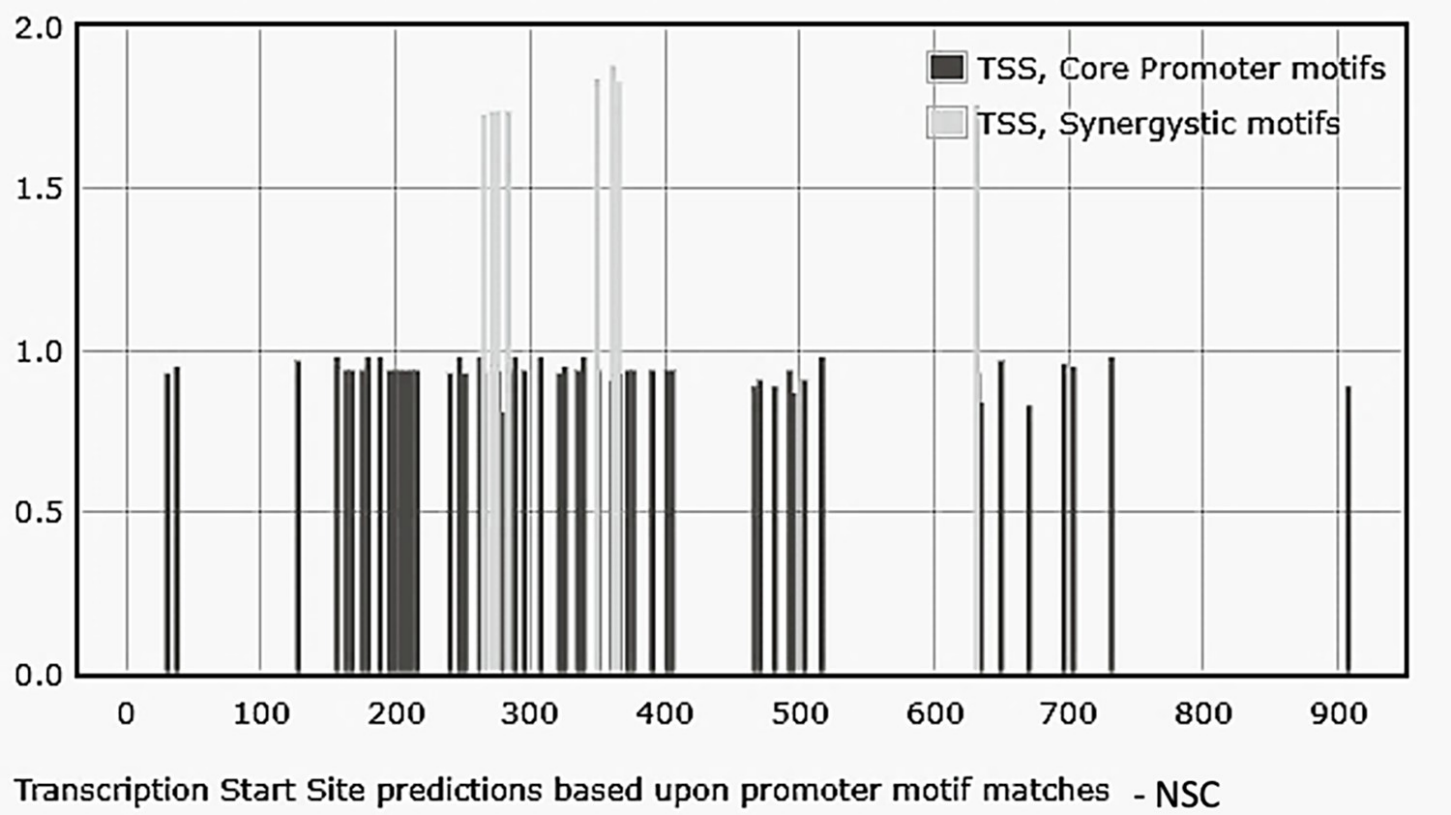
Profiling of the core promoter motifs identified in the upstream region of NANOGP8 DNA associated with NSC-derived exosomes. The sequences were analyzed using the YAPP Eukaryotic Core Promoter Predictor. Synergistic core promoter motifs are denoted in light gray, whereas the bars in dark grey depict individual motifs. **S1A and S1B Table** details the sequences and the type of promoter element.

Upstream NANOG DNA sequences associated with NSC-derived exosomes revealed forty promoter elements with regulatory protein-binding motifs, depending on TSS locations anticipated by the YAPP tool. Out of these, four were TATA boxes and the rest DPE. Seven synergistic combinations of the TATA box and DPE were also detected. Thirty-eight motifs had a matrix similarity score of >0.9, indicating a near-perfect match to a consensus nucleotide sequence within promoter elements. **Fig 5** and **S1A Table** detail the promoter element analysis. **S1B Table** lists the synergistic combination matches of the sequences and the type of promoter motifs of the NANOGP8 promoter region.

#### Analysis of the upstream sequence of NANOG DNA associated with GBM exosomes

**Fig 6:**
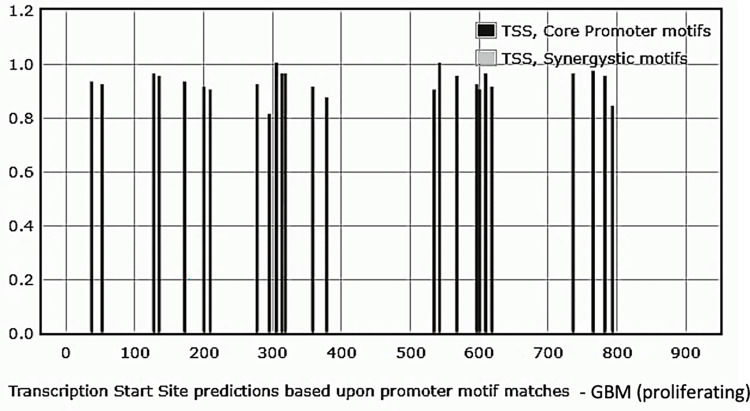
Profiling of the core promoter motifs identified in the upstream region of NANOGP8 DNA associated with GBM-derived exosomes. The sequences were analyzed using the YAPP Eukaryotic Core Promoter Predictor. The bars in dark grey depict individual motifs. No synergistic core promoter combination was detected. **S2 Table** details the sequences and the types of promoter elements.

The GBM exosomes are collected from the suspension culture of GBM neurospheres containing a mixed population of CD133^-^ cancer cells and CD133^+^ CSCs. **Fig 6** depicts the core promoter profiles. Out of twenty-four core promoters identified, five were INR, and the rest were DPE. No TATA box elements or synergistic combination matches of the sequences were detected.

#### Analysis of the upstream sequence of NANOG DNA associated with CD133^-^ GBM exosomes

**Fig 7:**
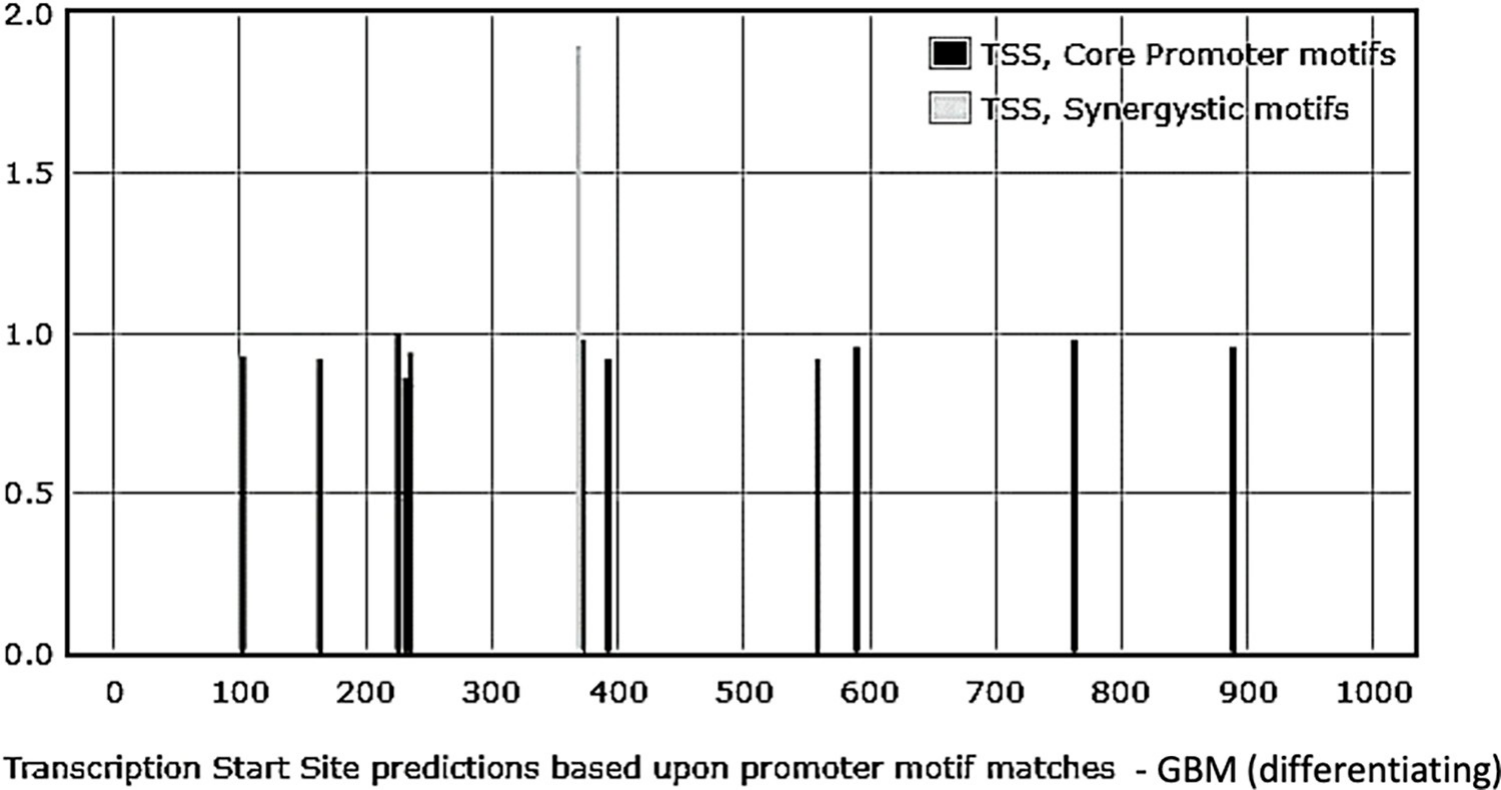
Profiling of the core promoter motifs identified in the upstream region of NANOGP8 DNA associated with CD133^-^ derived exosomes. The sequences were analyzed using the YAPP Eukaryotic Core Promoter Predictor. A singular synergistic core promoter motif is denoted in light gray, whereas the bars in dark grey depict individual motifs. **S3A and S3B Table** details the sequences and the type of promoter element.

Out of twelve core promoters identified, the upstream of the NANOG exosomal DNA sequence from CD133^-^ GBM had three INR and nine DPE core promoter elements. However, there were no TATA box elements similar to the sample from GBM exosomes. One synergistic motif combining INR and DPE elements was detected. **Fig 7** depicts the core promoter profiles and **S3A and S3B Table** details the sequences and the types of promoter elements.

### Viral sequence inundation analysis

**Fig 8:**
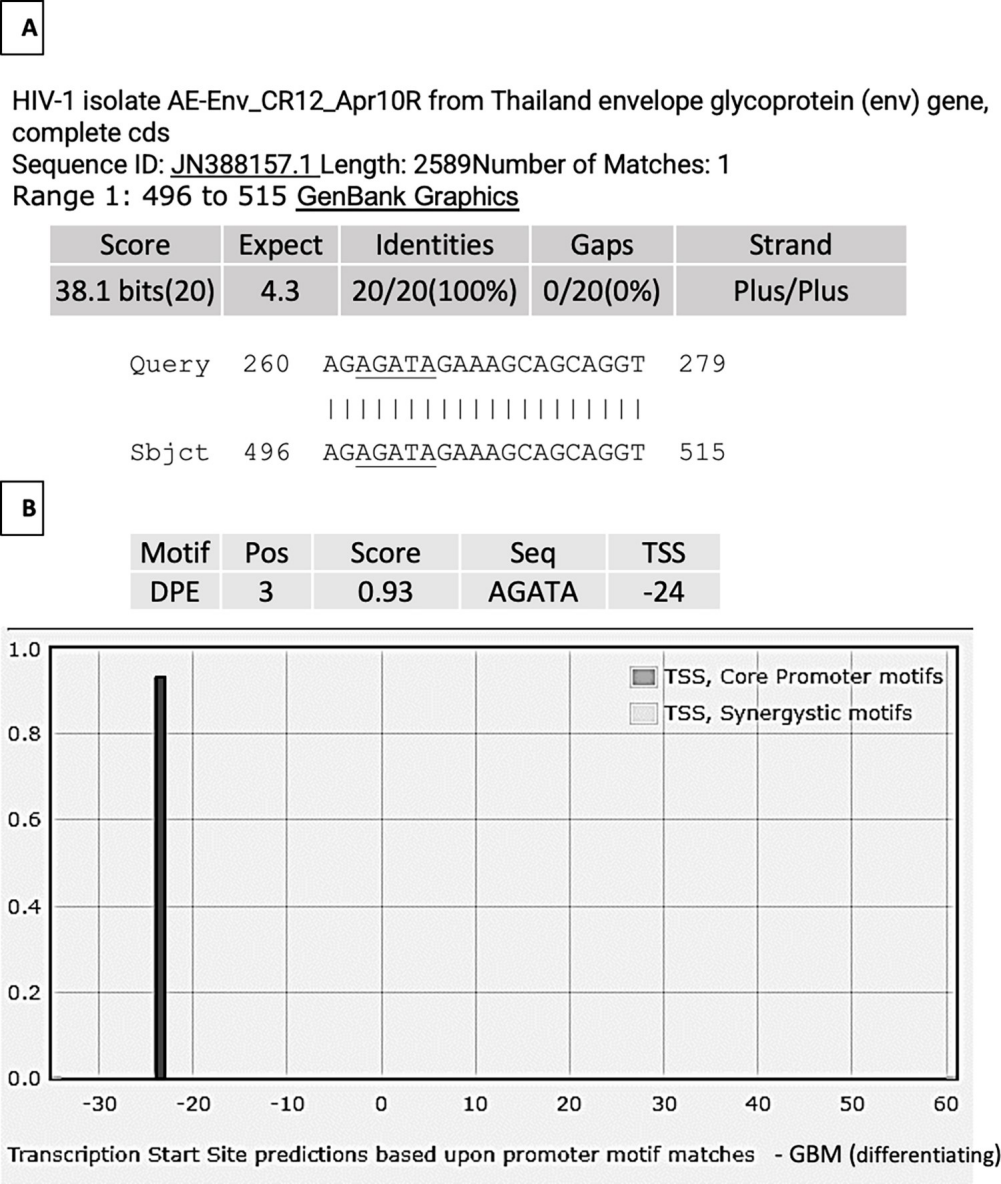
CD133^-^ GBM derived exosomal DNA sequence (partial). **(A)** 20 nucleotide identity to HIV-1 isolate AE-Env_CR12_Apr10R from Thailand envelope glycoprotein (env) gene (JN388157.1). **(B)** The nucleotides underlined in panel A show motif for a core promoter element DPE, in the HIV-1 sequence, detected by the YAPP tool. Retrovirus inserts in the upstream region may provide promoter sequences.

The upstream sequences of exosomal NANOGP8 DNA aligned with Human papillomavirus HPV (NC_027779), Epstein-Barr virus EBV (V01555), hepatitis B (KY417926), and Human T-cell leukemia virus type 1 or HTLV-1 (JX507077) showed no identity. The same exosomal sequences were also plugged into NCBI’s retrovirus nucleotide BLAST window to check for identities with a general retroviral sequence database. Exosomal DNA clone from CD133^-^ GBM showed identity to 20 nucleotides of human immunodeficiency virus HIV-1 isolate AE-Env_CR12_Apr10R from Thailand envelope glycoprotein (env) gene (JN388157.1) (**Fig 8A**). The underlined nucleotides “AGATA” denote promoter element DPE present in the viral sequence (**Fig 8B**). This result is an example of a possibility of retroviruses providing the promoter region with regulatory sequences.

Unexpectedly, all the upstream sequences of exosomal NANOGP8 DNA showed identity to Alcelaphine Herpesvirus-1, also known as Malignant Catarrhal Fever Virus (NC_002531), in varying degrees. This virus is not reported to infect humans. Human herpesviruses such as Gammaherpesvirus (NC_009333, NC_007605) and Human herpesvirus 4 type 2 (NC_009334) share a significant identity Alcelaphine Herpesvirus-1. Therefore, the exosomal sequences matching with Alcelaphine Herpesvirus-1 were aligned with the three human herpes viruses. Although human herpes viruses are implicated in high-grade gliomas, no significant similarities were detected [43].

#### Identities with retroviral elements

**Fig 9:**
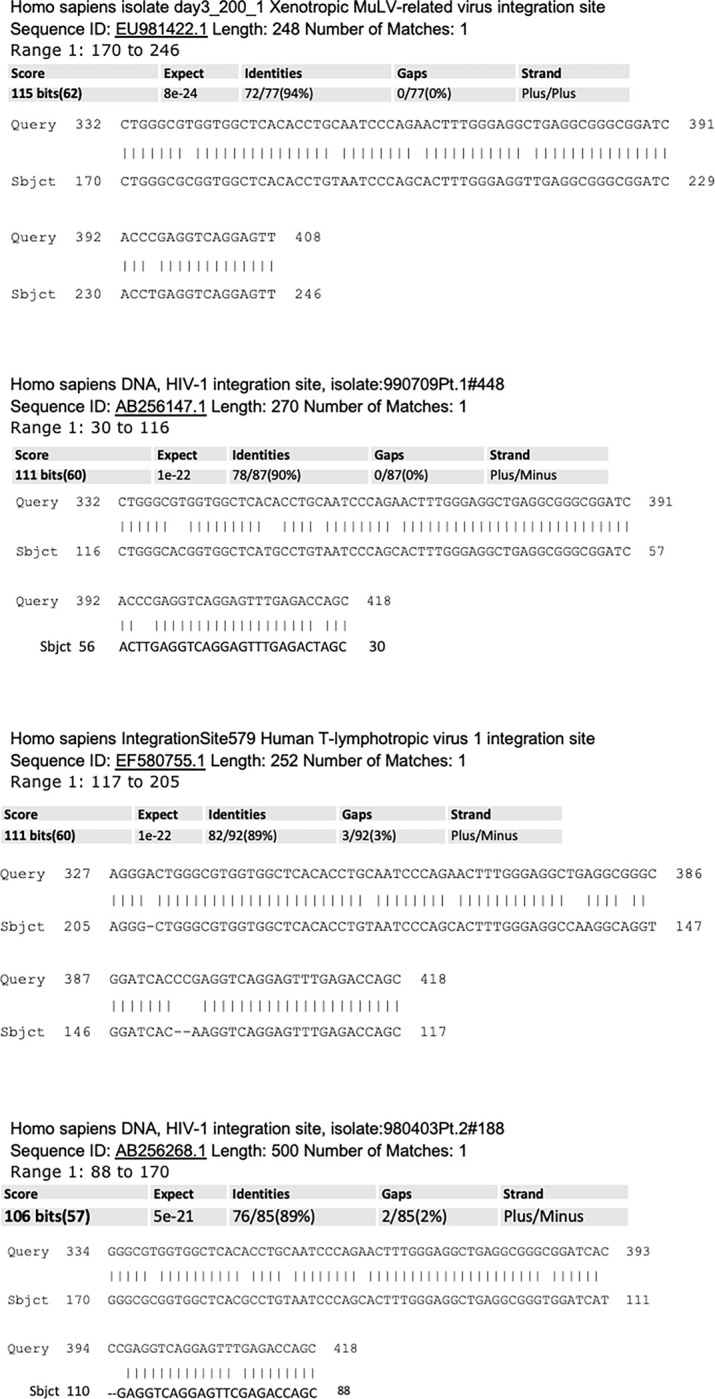
Standard BLAST analysis of NSC exosomal DNA clone after omitting the sequences for pCR4TOPO-TA vector and NANOG/NANOGP8 gDNA. The remaining sequence reveals the identities with various retroviral integration sites.

**Fig 10:**
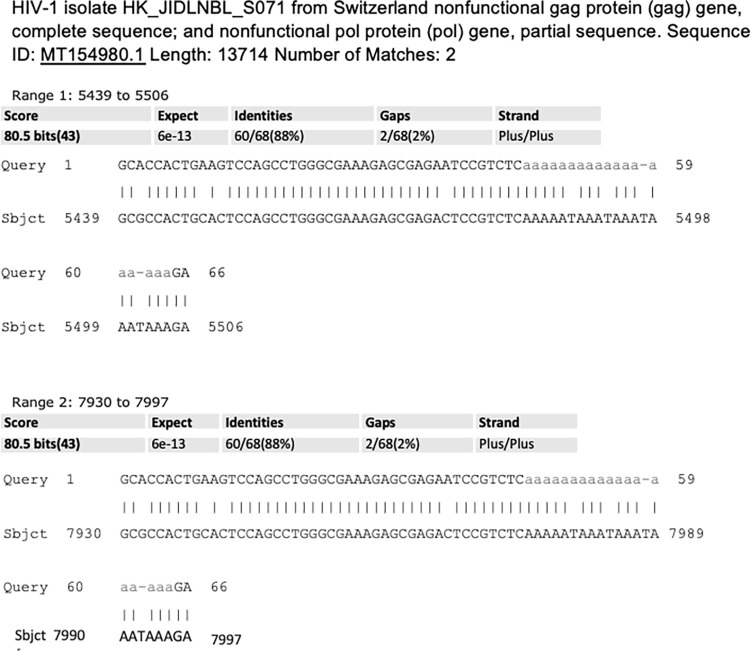
Standard BLAST analysis of GBM exosomal DNA clone after omitting the sequences identical to pCR4TOPO-TA vector and NANOG/NANOGP8 gDNA. The remaining sequence reveals the identities with HIV-1 genes.

Upstream sequences of exosomal NANOGP8 DNA of NSC, GBM and CSC showed identities to retrovirus gene sequences, retroviral LTRs, and their integration sites. NSC exosomes showed identities to Human Immunodeficiency Virus -1 (HIV-1), and Murine leukemia virus Graffi GV-1.4 (MuLV)—a gammaretrovirus, and its xenotropic integration site [44, 45]. The other identities included integration sites for HeLa-200, a retrovirus-like Human T-lymphotropic virus 1 as well as Homo sapiens Simian immunodeficiency virus. The upstream sequences of exosomal NANOGP8 DNA of GBM exosomes showed interesting results in standard BLAST. In addition to HIV-1 env and gag genes, the sequences matched with HIV-1 LTR and Human endogenous retrovirus HERV15 AZFa duplication junction LTR. The upstream sequences of exosomal NANOG DNA from CD133^-^ GBM exosomes revealed identity to HIV-1 strain GS66 LTR repeat region. In standard BLAST, the upstream sequences of exosomal NANOGP8 DNA of NSC matched with virus integration sites for MuLV, Human T-lymphotropic virus 1, and various isolates of HIV-1 **(Fig 9)**. GBM exosomal DNA clone had a match with HIV-1 isolate HK_JIDLNBL_S071 from Switzerland nonfunctional gag protein (gag) gene, complete sequence; and nonfunctional pol protein (pol) gene, partial sequence **(Fig 10)**. CD133^-^ GBM exosomal clone showed only one 20-nucleotide extended identity with HIV-1 isolate AE-Env_CR12_Apr10R from Thailand envelope glycoprotein (env) gene, complete cds as shown in **Fig 8(A)**.

**Fig 11:**
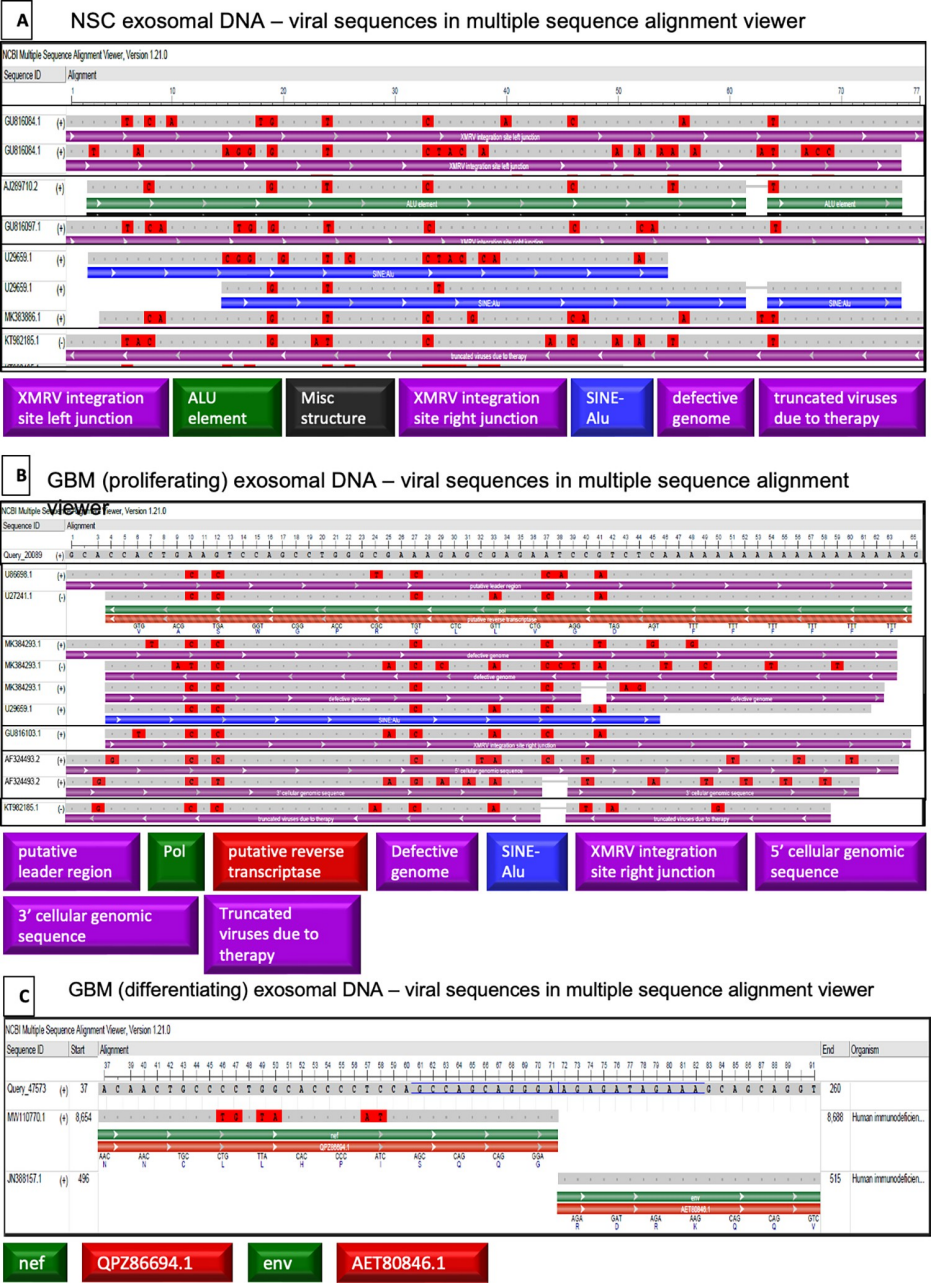
Analysis of the exosomal viral identities using Multiple Sequence Alignment Viewer 1.21.0. (A) NSC, (B) GBM—proliferating and (C) differentiating (CD133^-^) GBM exosomal DNA.

Using Multiple Sequence Alignment Viewer 1.21.0, the details of the viral identities were further analyzed. Importantly, exosomal DNA showed SINE-Alu elements in the sequences upstream of NANOG. Alu element inserted near a gene potentially influences its regulation with oncogenic implications [46]. **Fig 11** shows the details of the alignment.

### Putative transcription factor binding site (TFBS) analysis

Though some TFBS are shared by NSC, GBM as well as CD133^-^ GBM, a few are unique to each cell type. It is important to identify the significance of each of these TFBS because, in GBM, the upregulated TFs with binding sites in exosomal sequences may have a crucial role in NANOGP8 transcription [47]. **Table 2** summarizes the TFs for which the binding sites were found in the exosomal DNA.

#### Binding sites for TFs in NSC exosomes

As mentioned earlier, exosomal DNA clones of NSC had a unique pattern of NANOG and non-NANOG sequences alternating with each other. The non-NANOG sequences are considered for promoter analysis and mentioned as the first and second upstream sequences of exosomal NANOGP8 DNA. Binding sites for TFs MBF1, a member of the mediator protein family that connects general TF to gene-specific ones affecting the individual TF’s function and enhancing transcriptional efficacy was detected in the first upstream sequence of NSC. A binding site for another TF, EFII, was also present. The second upstream sequence of NSC exosomal DNA was found to have TFBS for signal transducer and activator of transcription proteins (STAT) family members. Their presence is significant because, through the conserved DNA binding domain, STAT TFs are the major players in transcription control in normal cells as well as CSCs, including GBM [48, 49]. Another interesting TF, YY1, has a binding site in the exosomal DNA of NSCs. YY1 is implicated in the transcriptional regulation of RSV [50]. The binding site for PEA3, another member of the ETS transcription factor family, was detected in NSC upstream sequences of exosomal NANOGP8 DNA. PEA3 subfamily members are known to promote stemness in CSC via NANOG and SOX2 transcription, thereby qualifying as an oncogenic transcription factor [51]. The presence of the binding sites for histone acetyltransferase (HAT) p300 is also significant because this TF is involved in the transcriptional upregulation of NANOG [52].

#### Binding sites for TFs in GBM and CD133^-^ GBM exosomes

The TFBS analysis of the upstream sequences of exosomal NANOGP8 DNA in GBM revealed a binding site for a solitary TF, myocyte enhancer factor 2A (MEF-2A). The presence of the binding sequence for this TF in exosomes secreted by GBM proliferating cells is compelling because MEF-2A is reported to promote stemness in gliomas, though limited research has found it to be through SOX2 transcription [53]. Cheng et al. have analyzed the GBM transcriptome for cancer-specific TFs to validate them as prognostic markers [47]. The researchers categorized the TFs based on their roles in certain cancer-specific pathways and clustered them accordingly. MEF-2A belonged to a cluster that includes stem cell proliferation in GBM. The patients with MEF-2A enrichment in signaling pathways for this cluster had the worst prognosis. Binding sites for the TFs Activator protein-1 or AP-1 and GA-binding protein- GABP were found in the exosomal DNA of CD133^-^ GBM cells. AP-1 is a transcription factor complex comprising hetero- or homodimerized leucine zipper proteins of FOS and JUN subfamilies [54]. Where c-Jun is implicated in the maintenance of glioma stem cells, its dimerization with c-Fos has a better DNA binding efficiency with the AP-1 DNA site [55, 56]. AP1 is shown to modulate NANOG activity in colorectal cancer stem cells as well [57]. c-Fos and cJun TFBS were found in all three types of exosomes. Similarly, GABP is another TF composed of a protein complex that regulates viral genes. Given that the NANOGP8 DNA found in exosomes has flanking retroviral sequences, the presence of GABP TFBS seems logical. GABP mediates protein-protein interaction of several TFs like Sp1, C/EBPα, and c-Myb which have binding sites in all three types of exosomal DNA [58]. Leukemia virus factor binding sites LVb and LVc found in MuLV have the consensus sequences for the binding of Ets family TFs [59]. MuLV sequence identity and TFBS LVb / LVc are found in GBM exosomal DNA. TFBS for another important immune response and inflammation regulatory protein, NF-κB, is detected exclusively in GBM exosomal DNA. NF-κB mediates oncogenic effects with implications in GBM CSC. The role of NF-κB in promoting GBM CSC is so involved that the GBM therapy drug Temozolomide efficacy can be improved by NF-κB inhibition [60]. Considering together, these facts possibly imply the role of NF-κB indirect regulation of NANOGP8 by controlling its transcription. A binding site for another TF, MBP-1, a Myc promoter binding protein, is detected in GBM exosomal DNA. MBP-1 is known to repress the transcription of multiple proto-oncogenes [61]. Given that TFBS for MBP-1 is present in exosomes derived from only the GBM exosomes, the role of TF in negatively regulating the NANOGP8 transcription needs to be investigated. The binding site for another transcription factor, neurofibromin 1 or NF-1 was found in GBM upstream sequences of exosomal NANOGP8 DNA. The presence of the binding site for this particular TF is interesting because, in the absence of the TATA box, NF-1 is required for basal transcription. e.g., NF-1 interaction with the basal promoter region is crucial for activating the TATA box-lacking type IIb sodium-phosphate cotransporters (NaPi-IIb) gene [62]. Incidentally, core promoter element analysis has revealed the absence of a TATA box in GBM-derived exosomal DNA. NF-1 is also known to transactivate viral promoters and nucleosome remodeling [63]. Nuclear hormone receptor- Glucocorticoid receptor (GR) binding sequence is also found in GBM-derived exosomal DNA. DNA-bound GR facilitates the formation of transcription initiation mega-complex involving other transcription factors such as p300 and CBP, for which the TFBS are found in exosomal DNA, in the vicinity of NANOG/NANOGP8 [64].

#### Binding sites for TFs common to NSC and GBM exosomes

YAP1 and POU2F1 binding sites were found in NSC as well as GBM exosomal DNA. Where the YAP1 binding site was detected in proliferating cells’ exosomes, the POU2F1 binding site was found in the exosomes secreted by the differentiating CD133^-^ cells. Yes-associated protein (YAP1) is a transcriptional regulator in the Hippo tumor suppressor signaling pathway. YAP binds to the promoters of many stemness genes, consequently regulating their expression [65–67]. Similarly, the detection of binding sites for POU2F1 (OCT1), a TF specifically binding to an octameric DNA sequence, is significant because the protein is implicated in the postsurgical invasion of glioma cells [68]. NANOGP8 is shown to have a role in EMT, and the POU2F1 may have a regulatory effect on it. Another important TFBS detected in the exosomal DNA of NSC and GBM is for chromatin remodeling ancillary transcription factor HMGI(Y). Highly expressed in embryonic and adult stem cells and inducing NANOG expression, HMGI (also known as HMGA1) is implicated in cancers [69, 70]. TFBS for some crucial transcription factors were detected in NANOGP8 exosomal DNA. If the TFs for which the binding sites detected in exosomal NANOGP8 DNA occupy their cogent sequences on the regulatory region, then they may have a crucial role in NANOGP8 transcription.

### Promoter analysis of gDNA clones

To compare the upstream sequences of exosomal NANOGP8 DNA with the nuclear DNA, we amplified the gDNA using NANOGP8 specific primers as described earlier. NSC and CD133^+^GBM-gDNA clones have 100% sequence identity **(S1 Fig)**. Therefore, the following promoter element analysis, TFBS analysis, and retrotransposable/retroviral inundation analysis results shown for the CD133^+^GBM-gDNA clone are valid for NSC as well.

#### Promoter element analysis

**Fig 12:**
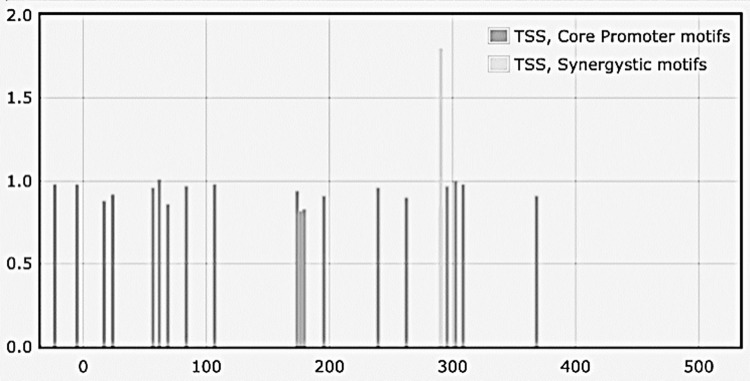
Core promoter motifs of the NANOGP8 promoter region in CD133^+^ GBM gDNA clones analyzed using the YAPP Eukaryotic Core Promoter Predictor.

Just like exosomal DNA analysis, the YAPP program was used to scan for canonical core promoter elements such as BREs, TATA boxes, INRs and DPEs, and their synergistic combinations in gDNA sequences (**Fig 12**). Along with several core promoter elements like TATA, INR, and DPE, a singular synergistic combination comprising TATA and INR motifs was detected (**S4A and S4B Table)**.

#### Retrovirus/Retrotransposable element identities

**Fig 13:**
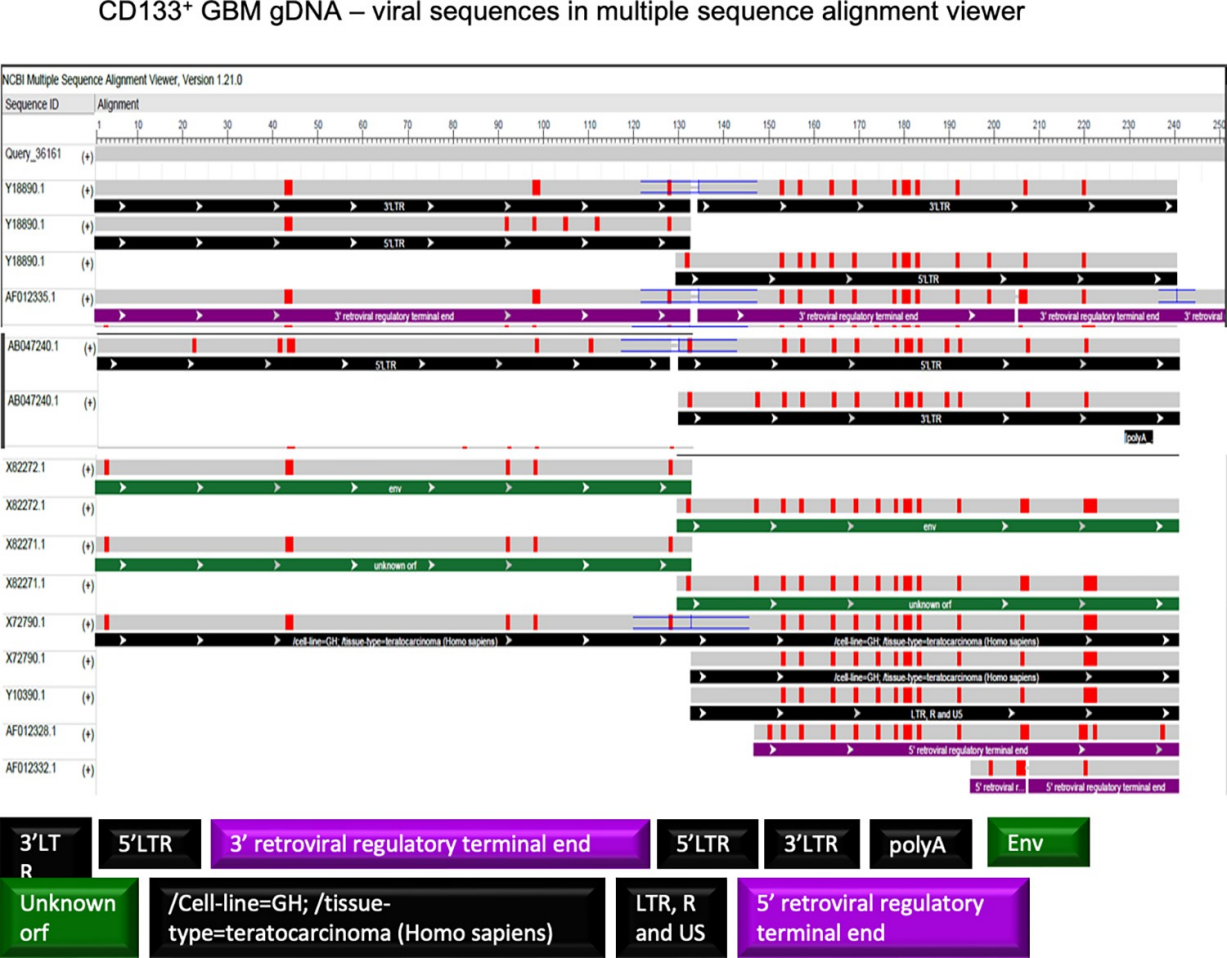
Analysis of the NSC and CD133^+^GBM-gDNA clone. Detection of viral identities using Multiple Sequence Alignment Viewer 1.21.0.

In the gDNA of CD133^+^GBM and NSC, out of 493 nucleotides analyzed for promoter region BLAST, 275 nucleotides directly flanking upstream of NANOGP8 UTR have a viral identity. The remaining 218 nucleotides show no identity to any known sequence in standard BLAST but show a 100% identity to NANOGP8 in human BLAST. The viral identities found in 275 nucleotide-long sequences are summarized in **Table 3**. Where exosomal DNA of NSC and GBM shows SINE-Alu sequence identities in multiple sequence alignment **(Fig 11)**, gDNA, lacking these identities, displays 5’ and 3’ retroviral regulatory terminal ends **(Fig 13)**. Although Kessler et al. have suggested no role of spliced HERV-K product in GBM samples they studied, the full-length transcripts are detected in many cancers, including GBM, with possible implications in oncogenesis [71].

#### NSC and CD133^+^GBM-gDNA clone-TFBS analysis

TFBS for HMGI(Y), STAT, YY1, NF-AT2, Elk-1, AP-1 (c-Jun, c-Fos), GR, POU2F1, E2F, c-Ets-1, and c-Ets-2 found in exosomal sequences are also detected in gDNA. The oncogenic and stem cell-related significance of the TFs has already been discussed in exosomal DNA analysis. TFs NF-AT1, NF-AT4, and NF-AT3 belong to the nuclear factor of activated T cells family that has a regulatory role in other cell types besides T lymphocytes. NFAT proteins are reported to regulate metastasis-related genes [72]. IRF-1 (interferon regulatory factor 1) TF is a tumor suppressor in cell proliferation and apoptosis. In the context of GBM, angiogenesis inhibitor bevacizumab-resistant tumors have increased IRF-1 signaling [73]. Similarly, as stated earlier, FOXN2 plays a suppressive role in the invasion and proliferation of human hepatocellular carcinoma cells. Together, suppresser and promoter TFs modulate the NANOGP8 expression. The list of all the TFBS found in gDNA clones has been given in **S5 Table**.

#### TFBS found in the upstream of NANOGP8 common in exosomal and gDNA of GBM, CD133^+^ GBM and NSC

Some of the TFBSs found in the upstream of NANOGP8 were common in exosomal as well as gDNA clones from *GBM*, *CD133*^*+*^ *GBM and NSC*. (**Table 4**). Especially, *c-Ets-1 and c-Ets-2 TFBS* were found in the sequences of exosomal DNA and CD133^+^ GBM gDNA sequences, respectively. Though the binding sites for many other TFs have been detected in the exosomal sequences, c-Ets-1 TFBS warrants special mention and further analysis. Not much research literature is available for the regulatory region analysis of the NANOGP8 gene. For the first time, Park et al. have analyzed transcription factor Fli-1 as a direct regulator of NANOGP8, identifying its putative binding site and functionality. Friend leukemia integration 1 (Fli-1), a proto-oncogene, is an ETS transcription factor with a E26 transformation-specific (ETS) binding domain that recognizes the core consensus sequence of GGAA/T in the promoter region of the target gene [74]. TFBS for c-Ets-1 found in exosomal DNA sequences is significant in this regard. Both, Fli-1 (Gene ID: 2313, updated on 12-Sep-2021 in NCBI database) and c-Ets-1 (gene ID: 2113, updated on 17-Oct-2021 in NCBI database), also known as EWSR2, share 98% homology in their ETS domain. Additionally, c-Ets-1 and c-Ets-2 interact with the c-Fos/c-Jun complex, acting as transcriptional regulators of cellular and viral genes [75]. Exosomal delivery of regulatory sequences for ETS domain flanking NANOGP8 may contribute to its transcription in GBM and NSC.

## Discussion

Glycoprotein antigen CD133 is expressed by normal as well as cancer stem cells [76]. It is essential for imparting cancer specific stemness to GBM cells [77]. Because NANOG, in the form of NANOGP8 expression, is required for the maintenance of CD133^+^ CSC, we used CD133 antibody for the immunocapture of CSC from the mixed population of GBM cells [20]. In our experiments, as GBM progresses from a CD133^+^ (undifferentiated) to a CD133^-^ (differentiated) state, NANOGP8 expression decreases significantly. Since GBM cells used in our analysis originated from the same subject, samples were genetically homologous. An epigenetic modification of the upstream region of the gene is most likely responsible for the changes in NANOGP8 expression. The role of exosomal NANOGP8 DNA remains to be elucidated in the alteration process.

GBM and NSC cell lines in our study have identical genomic sequences of the NANOGP8 upstream region. However, corresponding regions of their exosomal DNA shared no identities with the genomic sequences or the sequence reported by NCBI. Consequently, the core promoter element, viral sequence inundation, and the putative transcription factor binding site (TFBS) analyses of NANOGP8 exosomal DNA yielded different outcomes for each cell line. The exosomal DNA of NSC had a unique pattern. The BLAST analysis revealed that, unlike GBM, NSC exosomal DNA had NANOG/NANOGP8 sequences punctuated with non-NANOG stretches. The dissimilarities between exosomal DNA sequences and comparable genomic sequences suggest that exosomal NANOGP8 DNA with its upstream region is possibly acquired from a source other than the cell’s genome. An unexplored import mechanism may account for the existence of NANOGP8 DNA in the exosomes. The following paragraphs discuss the possible role of exosomal NANOGP8 regulatory sequences in transcription of this gene.

We have detected regulatory sequences comprising multiple core promoter elements and TFBS flanking the exosomal NANOGP8 DNA. The transcription factors (TFs) having binding sites in exosomal DNA are implicated in oncogenic regulation. The same region of exosomal DNA has also revealed cancer-related retrovirus sequences containing long terminal repeats (LTRs) known to modulate cellular oncogene expression [78]. Therefore, we postulate that exosomal NANOGP8 DNA, with its differential regulatory sequences, in the presence of suitable transcription factors, potentially facilitate a transient or a stable NANOGP8 expression. This, in turn, transforms a normal cell into a GBM CSC with a markedly upregulated transcription of NANOGP8. In a cancer environment, a dynamic reprogramming of the onco-transcriptional network may elicit a transient expression of the gene using the regulatory elements provided by the exosomal DNA.

The role of a limited promoter with minimal promoter elements is also possible in the transcriptional regulation of NANOGP8 exosomal DNA. Not much research literature is available for the regulatory region analysis of the NANOGP8 gene. For the first time, Park et al. have analyzed transcription factor Fli-1 as a direct regulator of NANOGP8, identifying its putative binding site and functionality. With a serial deletion of the promoter region, they have identified a “minimal promoter region” of NANOGP8 DNA that is needed for the Fli-1 to interact. The ETS transcription binding site in the minimal promoter region is essential for transcriptional control of NANOGP8 by Fli-1. Friend leukemia integration 1 (Fli-1), a proto-oncogene, is an ETS transcription factor with a E26 transformation-specific (ETS) binding domain that recognizes the core consensus sequence of GGAA/T in the promoter region of the target gene. c-Ets-1 and c-Ets-2 TFBS were found in the sequences of exosomal DNA and CD133^+^ GBM gDNA sequences, respectively. This important discovery suggests a probability of a minimal promoter region of NANOGP8 for other transcription factors as well [74].

Fairbanks et al. reported that in the human genome, the NANOGP8 gene is inserted in reverse orientation in the long terminal repeat (LTR) region of the SVA element belonging to the SINE-VNTR-Alu (SVA) class of retrotransposons. They suggested that the core promoter elements residing in the LTR region may promote the transcription of the gene [18]. However, our gDNA analysis shows identities of the NANOGP8 upstream region to LTR of HERV-K, regulatory terminal end of Human Endogenous Retrovirus IDDMK1,2–22 and HIV-1 partial coding DNA sequence (cds). Other studies have shown that retrogene can be transcribed via utilization of promoters assigned to the adjacent genes, or very rarely, inheritance of the promoter region of the parent gene [79, 80]. NANOGP8 does not inherit the promoter region from NANOG. The comparison of NCBI-reported genomic sequences of NANOG (NC_000012.12) and NANOGP8 (NC_000015.10) shows that the regulatory regions are non-identical. The retrotransposable elements present in the neighborhood regions of a retrogene are also known to promote transcription. Reverse transcribed proviral DNA of retroviruses containing the promoter, enhancer, and hormone induction regulatory signals within LTR are known to regulate retrogene transcriptions [81, 82]. Retroviral sequence analysis of exosomal DNA shows regulatory elements comprising core transcription factor segments as well as TFBS for the TFs that participate in LTR control. Exosomal DNA of GBM has TFBS for the TFs such as NF-κB, Ets-1, AP-1, and CBP. These TFs are implicated in regulation of HIV-1 LTR, where AP-1 and NF-κB physically interact to activate the transcription of the LTR [83]. Integration of HIV-1 proviral DNA into a genome confers malignant transformation susceptibility to the cell that is genetically modified because of the viral DNA integration [84]. In this context, the role of exosomal DNA carrying the viral sequences in the vicinity of NANOG/NANOGP8 needs further investigation. Due to the presence of regulatory elements, the HERV-specific LTRs are known to transcribe human genes [85]. This human-specific promoter activity, especially in the context of retro-oncogenes like NANOGP8, is a potential transcribing aid in creating precancerous or cancerous state of the cell. In the light of these findings, exosomal DNA identities to HIV and MuLV sequences are worth investigating further for their role as regulatory region components in NANOGP8 transcription.

TFs regulate the transcription not only by binding to their designated DNA sequences but also by physical interaction with other TFs. Detection of TFBS for the transcription factors that act synergistically is important because it helps to postulate their possible role in NANOGP8 transcription. The upstream sequences of exosomal NANOG DNA in our analyses are found to possess binding sites for such TFs. For example, NF-I and C/EBPβ are a part of a TF complex that shapes local chromatin architecture and are needed for chromatin accessibility maintenance in stimulation-induced TFs [86]. Similarly, AP-1 (c-Jun, c-Fos) are the strong transcriptional coactivators bringing about the interaction of other transcriptional coactivators like c-Ets-1 and c-Ets-2, the ETS domain-containing TFs that are known to directly bind to the minimal promoter of NANOGP8 [74, 75]. Ets-1 and SP1 are known to form a complex and Ets-1 is required for HIV-1 LTR transcription, another important point in the context of the NANOGP8 sequence being flanked by HIV-1 elements in exosomal DNA [87]. The presence of TFBS in exosomal DNA for all these TFs needs empirical validation.

The role of various cancer-specific TFs for which the TFBS are detected in exosomal NANOGP8 DNA should also be analyzed. Out of over 2000 TFs in human transcriptome, there are only a few that control the transcription of oncogenes involved in cancer initiation and sustenance. Notably, NANOGP8 promoter sequences found in exosomal DNA do possess binding sites for almost all the oncogenic TFs identified by Mees group [88]. For example, the TFBS for NF-κB, AP-1, a member of retinoid receptor—RXR-alpha that is involved in growth signals, has been detected in exosomal DNA. Moreover, some other TFs involved in the unlimited replication of a cell are ETS and E2F. Binding sites for these two TFs are found in exosomal DNA flanking NANOGP8. However, extensive research is needed to prove the activity of these TFs in NANOGP8 regulation. Because a gene transcription is considered stochastic rather than deterministic, a cancer cell’s microenvironment may control the transcription in a stimulus-specific manner [89, 90]. Moreover, the TFs act pleiotropically with several target genes and different TFs may control the regulation of a singular gene. Therefore, NANOGP8’s transcriptional regulation possibly depends upon the cancer-specific stimuli and the availability of transcription factors at a given time.

Genomewide analysis of core promoter elements suggests that a combination of two or three elements makes a more potent promoter as compared to a single weak element [91]. Analysis of exosomal DNA flanking the NANOGP8 has revealed the presence of core promoter and regulatory elements for basal transcription. A few core promoter elements coincide with retroviral inundations. Binding sites for crucial transcription factors known to play role in the transcription of oncogenes in general and NANOG transcription, in particular, are also detected. Where TATA, DPE, and their synergistic combinations are found in NSC exosomal DNA, in the absence of a TATA box, GBM exosomes possess the core promoter elements such as INR and DPE. TSS influences the motifs discovered in their vicinity for transcription initiation, a crucial factor in the prediction of core promoter elements. On the other hand, the position of the core promoter elements relative to a solitary and well-defined TSS is predetermined. The NANOGP8 sequence found in exosomal DNA did not show a unique singular TSS. Therefore, various TSS are anticipated by the YAPP Eukaryotic Core Promoter Predictor app’s algorithm. Accordingly, several promoter elements are predicted. Alternative transcriptional initiation or imprecise transcriptional initiation using an alternative TSS besides the optimal or a designated TSS also takes place in a cell [92]. In the context of NANOGP8 transcription, the possibility of surrogate transcription initiation via exosomal DNA participation is a possibility that needs validation.

CpG island methylation in promoters is an important mechanism for epigenetic regulation via transcription silencing [93]. The absence of CpG islands in the exosomal and genomic sequences that we analyzed is of vital importance in this regard. It also shows that epigenetic modification may not have been the reason behind the differential expression of NANOGP8 in GBM, CSCs, and NSCs.

We have amplified the exosomal DNA with an indiscriminate strand status. The nucleotide sequences reported here may belong to an ssDNA or a dsDNA. Cheng et al have shown that a plasma membrane-associated transcription system exists in cells. The cytoplasmic DNA and the resulting transcripts are implicated in oncogenesis [94]. If the regulatory elements of the exosomal DNA play a role in NANOGP8 transcription using DNA-dependent RNA polymerases, it is likely through such a transcription system that requires a dsDNA. Interestingly, Bai et al report novel ssDNA binding proteins participating in the regulation of specific genes in cancer cells [95]. In view of this fact, exosomal ssDNA may have a function prompting NANOGP8 transcription.

It is important to underscore the possibility that the NANOG DNA found in the exosomes may belong to other NANOG paralogs besides NANOGP8. However, when transcribed due to the presence of regulatory elements, NANOGP8 is the only paralogue with the ability to make the oncogenic protein. We initially speculated that the insertions of proviral DNA fragments provide regulatory elements with protein binding abilities to the genomic NANOGP8 upstream region. We believed that theoretically, exosomal DNA with the regulatory elements flanking NANOGP8 integrate into the genome, providing auxiliary cis-acting transcriptional elements. Therefore, internalization of exosomes conveying promoter elements and viral sequences may possess the potential to instigate a normal cell to express the undesirable NANOGP8 protein thereby creating a cancer niche. One of the possible delivery vehicles for the proviral DNA inserts in exosomal NANOGP8 is exosomes, the nanovesicles transporting a cargo of retrotransposon elements and a repertoire of tattered retroviral DNA [87]. In addition to the reverse transcribed DNA of retroviruses, exosome-mediated delivery of highly pathological DNA viruses may also contribute to promoter region modification of a retrogene [88]. With this background, we hypothesized that NANOGP8 gains high expression in CSCs by acquiring promotors, thereby altering the upstream sequence in the genome. However, upstream genomic sequences of NANOGP8 are completely identical in the GBM and NSC cell lines we studied. Their sequences also match with the sequence reported in GenBank (NCBI). Therefore, gDNA analysis of NANOGP8 upstream sequences does not support our initial theory that exosomal DNA insertion in genomic NANOGP8 would confer the promoter elements to the gene. Thus, the acquisition of somatic sequential alterations in the promoter region is not a mechanism of differential expression of NANOGP8 in these cells. Additionally, the exosomal DNA sequences flanking NANOGP8 are completely different than their genomic counterparts suggesting a non-genomic DNA source.

We believe that the differential promoter sequences of exosomal DNA, combined with the microenvironment of a tumor, and the availability of oncogenic transcription factors, may have an essential role in NANOGP8 expression. Internalization of such DNA-carrying exosomes by a target cell may potentially convert a normal or a cancer cell to a CSC. Moreover, exosomal NANOGP8, along with the flanking regulatory region, possibly provides ancillary copies of the gene to create more transcripts resulting in cancer progression and metastasis. The participation of specific exosomal DNA sequences for NANOGP8 expression needs to be investigated to validate these theories.

In addition to promoter elements, other regulatory elements in exosomal DNA, if inserted in upstream region of NANOGP8, although at a substantial distance, may confer the enhancer function to the region [96]. Because the enhancer elements can activate promoter transcription irrespective of the orientation of the regulatory elements, the internalization of exosomes possessing such distinct sequences may potentially set in motion the NANOGP8 transcription in a normal cell. The exosomal NANOGP8 DNA, unlike gDNA residing in the nucleus, is fragmented, possessing a transcriptional advantage due to promoter element inundations. In the context of GBM, transcriptional activation of NANOGP8 confers stemness to cancer cells and affects the downstream genes, e.g. ABCG2, a multigene drug transporter gene responsible for drug efflux [97]. This is an underlying cause of cancer stem cells becoming recalcitrant to chemotherapy. Our unpublished data has indicated a shift in ABCG2 and NANOGP8 transcriptional levels when NSC and GBM cells are cocultured. Because the cells are separated in a way where intercellular communication is only possible via nanovesicles, GBM exosomes could play a major role in the NANOGP8 transcription onset of NSC. Exosome-mediated NANOGP8 delivery, along with the transcriptional regulatory components may have a prominent, albeit undesirable role in the process.

## Conclusion

The exosomal DNA of NANOGP8 gene may have a promoter with differential sequences that can be activated depending on the tumor microenvironment and the availability of oncogenic TFs. Uptake and internalization of such exosomes may stimulate a normal or a cancer cell to become a CSC, even though their gDNA sequences remain identical. This study is only a snapshot of the promoter region showing regulatory elements in the exosomal DNA belonging to a NANOG family member. It is highly possible that there are more transcriptionally essential sequence motifs in the vicinity or farther away from the analyzed region that affect the NANOGP8 transcription. Nevertheless, studies to track the journey of NANOGP8 exosomal DNA through its unloading into a normal cell, and the subsequent fate leading up to its transcription are essential. They will help to understand the participation of exosome-associated promoter elements in cancer initiation, progression, and metastasis.

## Supporting information

S1 Fig CD133^+^ GBM and NSC gDNA clones- comparison with each other using BLAST. gDNA was PCR amplified using NANOGP8-specific reverse primer in 5’ UTR (NCBI Reference Sequence: NC_000015.10: 35085273–35085294). The forward primer sits in a sequence from the upstream region of the gene reported in the NCBI database (NCBI Reference Sequence: NC_000015.10: 35085802–35085783). The sequences from both the cell lines matched 100% with each other.
(PDF)

S2 Fig **(A) and (B)**. gDNA clones’ BLAST comparison with the reported sequences of the NANOGP8 gene (NC_000015.10) and the adjoining upstream region. gDNA was PCR amplified using NANOGP8-specific reverse primer in 5’ UTR (NCBI Reference Sequence: NC_000015.10: 35085273–35085294). The forward primer sits in a sequence from the upstream region of the gene reported in the NCBI database (NCBI Reference Sequence: NC_000015.10: 35085802–35085783). The sequences from **(A)** NSC and **(B)** CD133^+^ GBM showed 99% identity with the reported sequences of the NANOGP8 gene (NC_000015.10), and the adjoining upstream region.
(PDF)

S1 Table NSC- derived exosomal NANOGP8 upstream region sequences analyzed using YAPP Eukaryotic Core Promoter Predictor. (**A**) The sequences and the types of promoter motifs of the NANOGP8 upstream region. (**B**) Synergistic combination matches of the sequences and the type of promoter motifs.
(PDF)

S2 Table GBM-derived exosomal NANOGP8 upstream region sequences analyzed using YAPP Eukaryotic Core Promoter Predictor. The sequences and the types of promoter motifs of the NANOGP8 upstream region. No synergistic combination matches of the sequences was detected.
(PDF)

S3 Table CD133^-^ GBM—derived exosomal NANOGP8 upstream region sequences analyzed using YAPP Eukaryotic Core Promoter Predictor. (**A**) The sequences and the types of promoter motifs of the NANOGP8 upstream region. (**B**) Synergistic combination matches of the sequences and the type of promoter motifs.
(PDF)

S4 Table CD133^+^ GBM—derived genomic NANOGP8 DNA upstream region sequences analyzed using YAPP Eukaryotic Core Promoter Predictor. (**A**) The sequences and the types of promoter motifs of the NANOGP8 upstream region. (**B**) Synergistic combination matches of the sequences and the type of promoter motifs.
(PDF)

S5 Table The list of TFBS found in gDNA clones of NSC and CD133^+^ GBM. Using a virtual laboratory app, “PROMO” (version 3.2.0), the upstream sequences of genomic NANOG DNA were scanned for TFBSs (http://alggen.lsi.upc.es/cgi-bin/promo_v3/promo/promoinit.cgi?dirDB=TF_8.3). This program identified the putative binding sites and the TF proteins that bind to them in DNA sequences. The app uses TFBSs defined by the TRANSFAC® eukaryotic TF database (version 8.3).
(PDF)

S1 Raw image (PDF)

S2 Raw image (PDF)

S3 Raw image (PDF)

S4 Raw image (PDF)

S5 Raw image (PDF)
