# Supplementary material for: Analysis of regulatory sequences in exosomal DNA of NANOGP8
Source: PLoS One. 2023 Jan 25;18(1):e0280959. doi: 10.1371/journal.pone.0280959 (PMC9876286; doi:10.1371/journal.pone.0280959)
Supplement: S1 Fig — gDNA was PCR amplified using NANOGP8-specific reverse primer in 5’ UTR (NCBI Reference Sequence: NC_000015.10: 35085273–35085294). The forward primer sits in a sequence from the upstream region of the gene reported in the NCBI database (NCBI Reference Sequence: NC_000015.10: 35085802–35085783). The sequences from both the cell lines matched 100% with each other. (PDF) [file pone.0280959.s001.pdf]

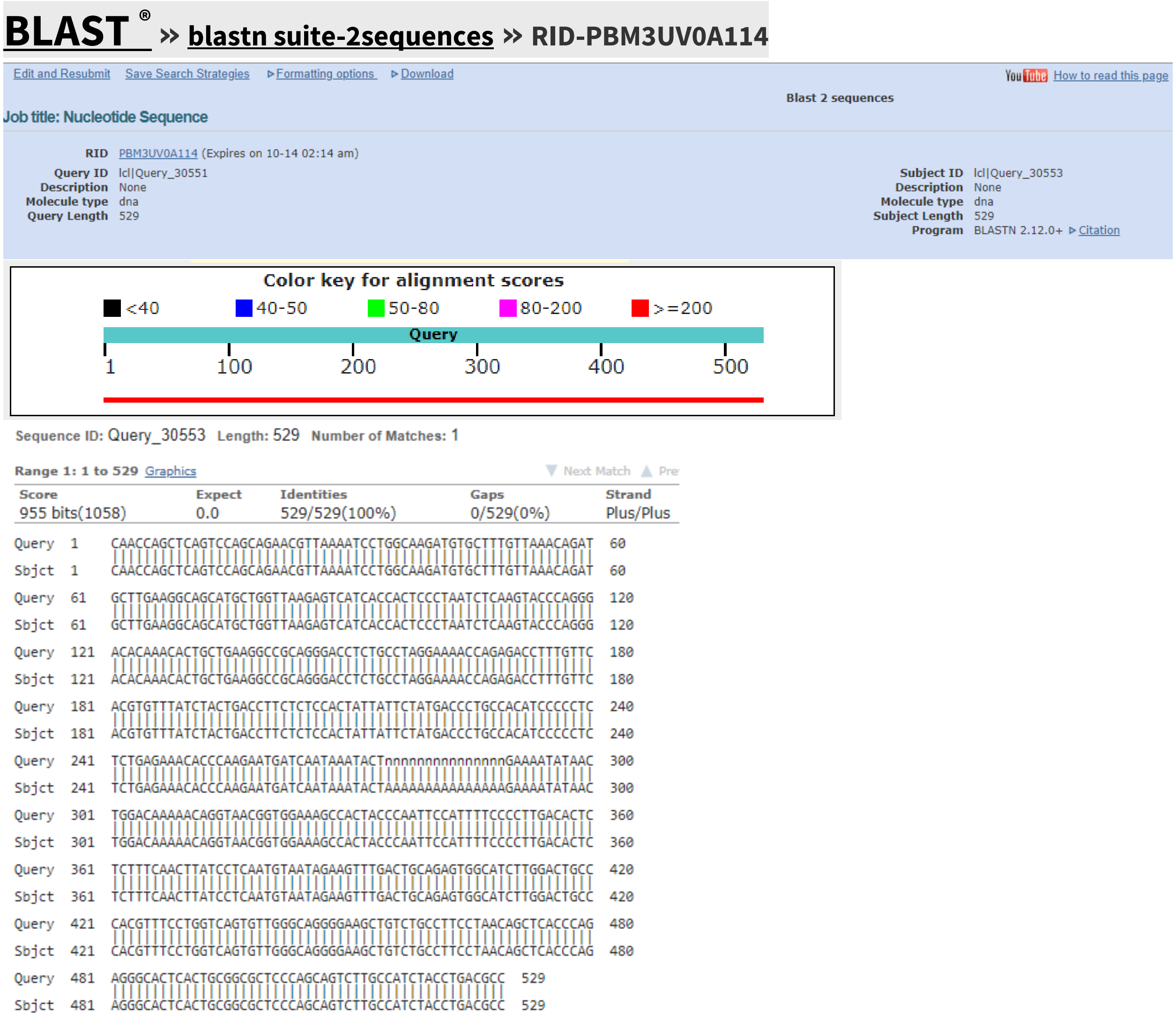

S1 Fig. CD133^{+} GBM and NSC gDNA clones- comparison with each other using BLAST. gDNA was PCR amplified using NANOGP8-specific reverse primer in 5' UTR (NCBI Reference Sequence: NC_000015.10: 35085273-35085294). The forward primer sits in a sequence from the upstream region of the gene reported in the NCBI database (NCBI Reference Sequence: NC_000015.10: 35085802-35085783). The sequences from both the cell lines matched 100% with each other.
