# Supplementary material for: Analysis of regulatory sequences in exosomal DNA of NANOGP8
Source: PLoS One. 2023 Jan 25;18(1):e0280959. doi: 10.1371/journal.pone.0280959 (PMC9876286; doi:10.1371/journal.pone.0280959)
Supplement: S2 Fig — (A) and (B). gDNA clones’ BLAST comparison with the reported sequences of the NANOGP8 gene (NC_000015.10) and the adjoining upstream region. gDNA was PCR amplified using NANOGP8-specific reverse primer in 5’ UTR (NCBI Reference Sequence: NC_000015.10: 35085273–35085294). The forward primer sits in a sequence from the upstream region of the gene reported in the NCBI database (NCBI Reference Sequence: NC_000015.10: 35085802–35085783). The sequences from (A) NSC and (B) CD133+ GBM showed 99% identity with the reported sequences of the NANOGP8 gene (NC_000015.10), and the adjoining upstream region. (PDF) [file pone.0280959.s002.pdf]

A

Job Title	**Nucleotide Sequence**
RID	N3N19UMT01N *Search expires on 09-28 22:24 pm* Download All
Program	BLASTN Citation
Database	Genome (GRCh38.p13 reference primary assembly top-level) See details
Query ID	lcl|Query_409877
Description	None
Molecule type	dna
Query Length	529
Other reports	Distance tree of results MSA viewer

Download GenBank Graphics

Homo sapiens chromosome 15, GRCh38.p13 Primary Assembly

Sequence ID: NC_000015.10 **Length:** 101991189 **Number of Matches:** 1

Range 1: 35085273 to 35085802 GenBank Graphics ▼ Next Match ▲ Previous Match

Score	Expect	Identities	Gaps	Strand
966 bits(523)	0.0	528/530(99%)	1/530(0%)	Plus/Plus

Features: 163 bp at 5' side: homeobox protein nanogp8
134715 bp at 3' side: diphthine--ammonia ligase isoform x2

```
Query  1         CAACCAGCTCAGTCCAGCAGAACGTTAAAATCCTGgcaagatgtgctttgttaaacagat  60
                 ||||||||||||||||||||||||||||||||||||||||||||||||||||||||||||
Sbjct  35085273  CAACCAGCTCAGTCCAGCAGAACGTTAAAATCCTGGCAAGATGTGCTTTGTTAAACAGAT  35085332

Query  61        gcttgaaggcagcatgctggttaagagtcatcaccactccctaatctcaagtacccaggg  120
                 ||||||||||||||||||||||||||||||||||||||||||||||||||||||||||||
Sbjct  35085333  GCTTGAAGGCAGCATGCTGGTTAAGAGTCATCACCACTCCCTAATCTCAAGTACCCAGGG  35085392

Query  121       acacaaacactgctgaaggccgcagggacctctgcctaggaaaaccagagacctttgttc  180
                 ||||||||||||||||||||||||||||||||||||||||||| ||||||||||||||||
Sbjct  35085393  ACACAAACACTGCTGAAGGCCGCAGGGACCTCTGCCTAGGAAAGCCAGAGACCTTTGTTC  35085452

Query  181       acgtgtttatctactgaccttctctccactattattctatgaccctgccacatcccctc  240
                 ||||||||||||||||||||||||||||||||||||||||||||||||||||||||||||
Sbjct  35085453  ACGTGTTTATCTACTGACCTTCTCTCCACTATTATTCTATGACCCTGCCACATCCCCCTC  35085512

Query  241       tctgagaaacacccaagaatgatcaataaatact-aaaaaaaaaaaaaaaGAAAATATAA  299
                 |||||||||||||||||||||||||||||||||| |||||||||||||||||||||||||
Sbjct  35085513  TCTGAGAAACACCCAAGAATGATCAATAAATACTAAAAAAAAAAAAAAAAGAAAATATAA  35085572

Query  300       CTGGACAAAAACAGGTAACGGTGGAAAGCCACTACCCAATTCCATTTTCCCCTTGACACT  359
                 ||||||||||||||||||||||||||||||||||||||||||||||||||||||||||||
Sbjct  35085573  CTGGACAAAAACAGGTAACGGTGGAAAGCCACTACCCAATTCCATTTTCCCCTTGACACT  35085632

Query  360       CTCTTTCAACTTATCCTCAATGTAATAGAAGTTTGACTGCAGAGTGGCATCTTGGACTGC  419
                 ||||||||||||||||||||||||||||||||||||||||||||||||||||||||||||
Sbjct  35085633  CTCTTTCAACTTATCCTCAATGTAATAGAAGTTTGACTGCAGAGTGGCATCTTGGACTGC  35085692

Query  420       CCACGTTTCCTGGTCAGTGTTGGGCAGGGGAAGCTGTCTGCCTTCCTAACAGCTCACCCA  479
                 ||||||||||||||||||||||||||||||||||||||||||||||||||||||||||||
Sbjct  35085693  CCACGTTTCCTGGTCAGTGTTGGGCAGGGGAAGCTGTCTGCCTTCCTAACAGCTCACCCA  35085752

Query  480       GAGGGCACTCACTGCGGCGCTCCCAGCAGTCTTGCCATCTACCTGACGCC  529
                 ||||||||||||||||||||||||||||||||||||||||||||||||||
Sbjct  35085753  GAGGGCACTCACTGCGGCGCTCCCAGCAGTCTTGCCATCTACCTGACGCC  35085802
```

B

Job Title	Nucleotide Sequence
RID	1TRKWTUH013 *Search expires on 03-01 21:38 pm* Download All
Program	BLASTN Citation
Database	Genome (GRCh38.p13 reference primary assembly top-level) See details
Query ID	lcl|Query_58819
Description	None
Molecule type	dna
Query Length	529
Other reports	Distance tree of results MSA viewer

Homo sapiens chromosome 15, GRCh38.p13 Primary Assembly

Sequence ID: NC_000015.10 Length: 101991189 Number of Matches: 103

Range 1: 35085273 to 35085802 GenBank Graphics ▼ Next Match ▲ Previous M

Score	Expect	Identities	Gaps	Strand
944 bits(1046)	0.0	528/530(99%)	1/530(0%)	Plus/Plus

Features: 163 bp at 5' side: homeobox protein nanogp8
134715 bp at 3' side: diphthine--ammonia ligase isoform x2

```
Query  1         CAACCAGCTCAGTCCAGCAGAACGTTAAAATCCTGGCAAGATGTGCTTTGTTAAACAGAT  60
                 ||||||||||||||||||||||||||||||||||||||||||||||||||||||||||||
Sbjct  35085273  CAACCAGCTCAGTCCAGCAGAACGTTAAAATCCTGGCAAGATGTGCTTTGTTAAACAGAT  35085332

Query  61        GCTTGAAGGCAGCATGCTGGTTAAGAGTCATCACCACTCCCTAATCTCAAGTACCCAGGG  120
                 ||||||||||||||||||||||||||||||||||||||||||||||||||||||||||||
Sbjct  35085333  GCTTGAAGGCAGCATGCTGGTTAAGAGTCATCACCACTCCCTAATCTCAAGTACCCAGGG  35085392

Query  121       ACACAAACACTGCTGAAGGCCGCAGGGACCTCTGCCTAGGAAAACCAGAGACCTTTGTTC  180
                 ||||||||||||||||||||||||||||||||||||||||||| ||||||||||||||||
Sbjct  35085393  ACACAAACACTGCTGAAGGCCGCAGGGACCTCTGCCTAGGAAAGCCAGAGACCTTTGTTC  35085452

Query  181       ACGTGTTTATCTACTGACCTTCTCTCCACTATTATTCTATGACCCTGCCACATCCCCCTC  240
                 ||||||||||||||||||||||||||||||||||||||||||||||||||||||||||||
Sbjct  35085453  ACGTGTTTATCTACTGACCTTCTCTCCACTATTATTCTATGACCCTGCCACATCCCCCTC  35085512

Query  241       TCTGAGAAACACCCAAGAATGATCAATAAATAct-aaaaaaaaaaaaaaagaaaataTAA  299
                 ||||||||||||||||||||||||||||||||| ||||||||||||||||||||||||||
Sbjct  35085513  TCTGAGAAACACCCAAGAATGATCAATAAATACTAAAAAAAAAAAAAAAAGAAAATATAA  35085572

Query  300       CTGGACAAAAACAGGTAACGGTGGAAAGCCACTACCCAATTCCATTTTCCCCTTGACACT  359
                 ||||||||||||||||||||||||||||||||||||||||||||||||||||||||||||
Sbjct  35085573  CTGGACAAAAACAGGTAACGGTGGAAAGCCACTACCCAATTCCATTTTCCCCTTGACACT  35085632

Query  360       CTCTTTCAACTTATCCTCAATGTAATAGAAGTTTGACTGCAGAGTGGCATCTTGGACTGC  419
                 ||||||||||||||||||||||||||||||||||||||||||||||||||||||||||||
Sbjct  35085633  CTCTTTCAACTTATCCTCAATGTAATAGAAGTTTGACTGCAGAGTGGCATCTTGGACTGC  35085692

Query  420       CCACGTTTCCTGGTCAGTGTTGGGCAGGGGAAGCTGTCTGCCTTCCTAACAGCTCACCCA  479
                 ||||||||||||||||||||||||||||||||||||||||||||||||||||||||||||
Sbjct  35085693  CCACGTTTCCTGGTCAGTGTTGGGCAGGGGAAGCTGTCTGCCTTCCTAACAGCTCACCCA  35085752

Query  480       GAGGGCACTCACTGCGGCGCTCCCAGCAGTCTTGCCATCTACCTGACGCC  529
                 ||||||||||||||||||||||||||||||||||||||||||||||||||
Sbjct  35085753  GAGGGCACTCACTGCGGCGCTCCCAGCAGTCTTGCCATCTACCTGACGCC  35085802
```

S2 Fig (A) and (B). gDNA clones' BLAST comparison with the reported sequences of the NANOGP8 gene (NC_000015.10) and the adjoining upstream region. gDNA was PCR amplified using NANOGP8-specific reverse primer in 5' UTR (NCBI Reference Sequence: NC_000015.10: 35085273-35085294). The forward primer sits in a sequence from the upstream region of the gene reported in the NCBI database (NCBI Reference Sequence: NC_000015.10: 35085802-35085783). The sequences from **(A)** NSC and **(B)** CD133^{+} GBM showed 99% identity with the reported sequences of the NANOGP8 gene (NC_000015.10), and the adjoining upstream region.
