# Supplementary material for: Analysis of regulatory sequences in exosomal DNA of NANOGP8
Source: PLoS One. 2023 Jan 25;18(1):e0280959. doi: 10.1371/journal.pone.0280959 (PMC9876286; doi:10.1371/journal.pone.0280959)
Supplement: S1 Table — (A) The sequences and the types of promoter motifs of the NANOGP8 upstream region. (B) Synergistic combination matches of the sequences and the type of promoter motifs. (PDF) [file pone.0280959.s003.pdf]

(A)

Motif	Pos	Score	Seq	TSS
TATA	112	0.8	ATAATATAGAAA	147
TATA	299	0.88	TAGATAAATAAA	334
TATA	193	0.9	TACATAAATAGA	228
TATA	303	0.9	TAAATAAATGAC	338
DPE	261	0.92	GGATA	234
DPE	161	0.92	GGATA	134
DPE	146	0.92	GGATA	119
DPE	216	0.92	GGATA	189
DPE	135	0.92	GGATA	108
DPE	271	0.93	AGATA	244
DPE	285	0.93	AGATA	258
DPE	230	0.93	AGATA	203
DPE	102	0.93	AGATA	75
DPE	98	0.93	AGATA	71
DPE	106	0.93	AGATA	79
DPE	94	0.93	AGATA	67
DPE	267	0.93	AGATA	240
DPE	296	0.93	AGATA	269
DPE	190	0.93	AGATA	163
DPE	90	0.93	AGATA	63
DPE	179	0.93	AGATA	152
DPE	110	0.93	AGATA	83
DPE	70	0.93	AGATA	43
DPE	300	0.93	AGATA	273
DPE	62	0.93	AGATA	35
DPE	58	0.93	AGATA	31
DPE	245	0.93	AGATA	218
DPE	167	0.93	AGATA	140
DPE	171	0.93	AGATA	144
DPE	220	0.94	AGATC	193
DPE	22	0.96	GGATG	-5
DPE	257	0.97	AGATG	230
DPE	74	0.97	AGATG	47
DPE	51	0.97	AGATG	24
DPE	202	0.97	AGATG	175
DPE	157	0.97	AGATG	130
DPE	183	0.97	AGATG	156
DPE	234	0.97	AGATG	207
DPE	83	0.97	AGATG	56
DPE	142	0.97	AGATG	115

(B)

Motif	Pos	Seq	Motif	Pos	Seq	Combined Score	TSS
TATA	112	ATAATATAGAAA	DPE	161	GGATA	1.72	133
TATA	112	ATAATATAGAAA	DPE	167	AGATA	1.73	139
TATA	112	ATAATATAGAAA	DPE	171	AGATA	1.73	143
TATA	112	ATAATATAGAAA	DPE	179	AGATA	1.73	151
TATA	193	TACATAAATAGA	DPE	245	AGATA	1.83	217
TATA	193	TACATAAATAGA	DPE	257	AGATG	1.87	229
TATA	193	TACATAAATAGA	DPE	261	GGATA	1.82	233

S1 Table. NSC- derived exosomal NANOGP8 upstream region sequences analyzed using YAPP Eukaryotic Core Promoter Predictor. (**A**) The sequences and the types of promoter motifs of the NANOGP8 upstream region. (**B**) Synergistic combination matches of the sequences and the type of promoter motifs.
