# Supplementary material for: Analysis of regulatory sequences in exosomal DNA of NANOGP8
Source: PLoS One. 2023 Jan 25;18(1):e0280959. doi: 10.1371/journal.pone.0280959 (PMC9876286; doi:10.1371/journal.pone.0280959)
Supplement: S2 Table — The sequences and the types of promoter motifs of the NANOGP8 upstream region. No synergistic combination matches of the sequences was detected. (PDF) [file pone.0280959.s004.pdf]

Motif	Pos	Score	Seq	TSS
INR	169	1	CCAGTCC	172
INR	808	1	CCAATCC	811
INR	242	0.87	CCAGACT	245
INR	657	0.84	CCAGATG	660
INR	159	0.81	CCAGAAG	162
DPE	436	1	AGACG	409
DPE	659	0.97	AGATG	632
DPE	21	0.96	GGATG	-6
DPE	212	0.96	GGATG	185
DPE	503	0.96	GGACC	476
DPE	207	0.96	AGACA	180
DPE	630	0.96	AGACA	603
DPE	461	0.95	GGACA	434
DPE	676	0.95	GGACA	649
DPE	29	0.95	GGACA	2
DPE	66	0.93	AGATA	39
DPE	490	0.92	AGTTG	463
DPE	171	0.92	AGTCC	144
DPE	512	0.91	GGTCC	485
DPE	94	0.91	GGTCC	67
DPE	252	0.91	GGTCC	225
DPE	103	0.9	GGTCA	76
DPE	428	0.9	GGTCA	401
DPE	494	0.9	GGTCA	467

S2 Table. GBM-derived exosomal NANOGP8 upstream region sequences analyzed using YAPP Eukaryotic Core Promoter Predictor. The sequences and the types of promoter motifs of the NANOGP8 upstream region. No synergistic combination matches of the sequences was detected.
