# Supplementary material for: Analysis of regulatory sequences in exosomal DNA of NANOGP8
Source: PLoS One. 2023 Jan 25;18(1):e0280959. doi: 10.1371/journal.pone.0280959 (PMC9876286; doi:10.1371/journal.pone.0280959)
Supplement: S3 Table — (A) The sequences and the types of promoter motifs of the NANOGP8 upstream region. (B) Synergistic combination matches of the sequences and the type of promoter motifs. (PDF) [file pone.0280959.s005.pdf]

(**A**)

Motif	Pos	Score	Seq	TSS
INR	203	0.99	CCATTTC	206
INR	350	0.97	TCATTTC	353
INR	209	0.85	CTAGTCT	212
DPE	770	0.97	AGATG	743
DPE	597	0.95	GGACA	570
DPE	896	0.95	GGACA	869
DPE	243	0.93	AGATA	216
DPE	110	0.92	AGTCC	83
DPE	400	0.91	GGTTG	373
DPE	378	0.91	GGTTG	351
DPE	171	0.91	GGTCC	144
DPE	566	0.91	AGTCA	539

(**B**)

Motif	Pos	Seq	Motif	Pos	Seq	Combined Score	TSS
INR	350	TCATTTC	DPE	378	GGTTG	1.89	350

S3 Table. CD133^{-} GBM - derived exosomal NANOGP8 upstream region sequences analyzed using YAPP Eukaryotic Core Promoter Predictor. (**A**) The sequences and the types of promoter motifs of the NANOGP8 upstream region. (**B**) Synergistic combination matches of the sequences and the type of promoter motifs.
