# Supplementary material for: Analysis of regulatory sequences in exosomal DNA of NANOGP8
Source: PLoS One. 2023 Jan 25;18(1):e0280959. doi: 10.1371/journal.pone.0280959 (PMC9876286; doi:10.1371/journal.pone.0280959)
Supplement: S4 Table — (A) The sequences and the types of promoter motifs of the NANOGP8 upstream region. (B) Synergistic combination matches of the sequences and the type of promoter motifs. (PDF) [file pone.0280959.s006.pdf]

(**A**)

Motif	Pos	Score	Seq	TSS
INR	58	1	CCACTCC	61
INR	299	0.99	CCAATTC	302
INR	305	0.97	CCATTTT	308
INR	292	0.96	CCACTAC	295
INR	170	0.93	CCACTAT	173
INR	192	0.9	CCACATC	195
INR	65	0.85	CTAATCT	68
INR	176	0.82	TTATTCT	179
INR	173	0.81	CTATTAT	176
TATA	227	0.89	TCAATAAATACT	262
TATA	255	0.83	AAAATATAACTG	290
DPE	3	0.97	AGATG	-24
DPE	21	0.97	AGATG	-6
DPE	133	0.97	AGACC	106
DPE	110	0.96	GGACC	83
DPE	266	0.95	GGACA	239
DPE	83	0.95	GGACA	56
DPE	50	0.91	AGTCA	23
DPE	395	0.9	GGTCA	368
DPE	43	0.87	GGTTA	16

(**B**)

Motif	Pos	Seq	Motif	Pos	Seq	Combined Score	TSS
TATA	255	AAAATATAACTG	INR	292	CCACTAC	1.79	290

S4 Table. CD133$^+$ GBM - derived genomic NANOGP8 DNA upstream region sequences analyzed using YAPP Eukaryotic Core Promoter Predictor. (**A**) The sequences and the types of promoter motifs of the NANOGP8 upstream region. (**B**) Synergistic combination matches of the sequences and the type of promoter motifs.
