# Supplementary material for: Analysis of regulatory sequences in exosomal DNA of NANOGP8
Source: PLoS One. 2023 Jan 25;18(1):e0280959. doi: 10.1371/journal.pone.0280959 (PMC9876286; doi:10.1371/journal.pone.0280959)
Supplement: S5 Table — Using a virtual laboratory app, “PROMO” (version 3.2.0), the upstream sequences of genomic NANOG DNA were scanned for TFBSs (http://alggen.lsi.upc.es/cgi-bin/promo_v3/promo/promoinit.cgi?dirDB=TF_8.3). This program identified the putative binding sites and the TF proteins that bind to them in DNA sequences. The app uses TFBSs defined by the TRANSFAC® eukaryotic TF database (version 8.3). (PDF) [file pone.0280959.s007.pdf]

	Transcription factors for which the binding sites are found in genomic DNA clones							
**gDNA: NSC and CD133^{+} GBM**	C/EBPbeta	LEF-1	TFIID	FOXP3	PR B	PR A	XBP-1	GR-alpha
	AP-2alphaA	AP-1	c-Jun	c-Fos	T3R-beta1	TFII-I	GR-beta	RXR-alpha
	EBF	GR	E2F-1	c-Ets-2	IRF-1	GR	NF-AT1	VDR
	c-Myc	USF1	HNF-3beta	GATA-1	GATA-1	ER-alpha	Crx	POU1F1a
	NF-1/L	c-Ets-1 68	PEA3	NFI/CTF	MEF-2A	HNF-3alpha	HOXD8	HOXD8
	POU1F1b	POU1F1c	PU.1	HNF-1C	HNF-1B	HOXD9	HOXD10	HOXD9
	HOXD9	HOXD10	HOXD10	c-Myb	STAT4	c-Ets-1	YY1	NF-AT2
	NF-AT1	RelA	STAT1beta	c-Jun	ENKTF-1	NF-1	Pax-5	p53
	Elk-1	HNF-1A	c-Jun	GCF	LyF-1	C/EBPalpha	AP-2	Elk-1
	NF-1	c-Ets-1	C/EBPalpha	NF-Y	NF-Y	NF-AT1	CAC-binding protein	AR
	HNF-4alpha1	VDR	PXR-1:RXR-alpha	SRY	TCF-4E	HNF-4alpha	LXR-alpha:RXR-alpha	HNF-1A
	c-Fos	C/EBPbeta						

S5 Table. The list of TFBS found in gDNA clones of NSC and CD133^{+} GBM. Using a virtual laboratory app, “PROMO” (version 3.2.0), the upstream sequences of genomic NANOG DNA were scanned for TFBSs (http://alggen.lsi.upc.es/cgi-bin/promo_v3/promo/promoinit.cgi?dirDB=TF_8.3). This program identified the putative binding sites and the TF proteins that bind to them in DNA sequences. The app uses TFBSs defined by the TRANSFAC® eukaryotic TF database (version 8.3).
