# Supplementary material for: Analysis of regulatory sequences in exosomal DNA of NANOGP8
Source: PLoS One. 2023 Jan 25;18(1):e0280959. doi: 10.1371/journal.pone.0280959 (PMC9876286; doi:10.1371/journal.pone.0280959)
Supplement: S1 Raw image — (PDF) [file pone.0280959.s008.pdf]

Raw image for Fig 2-A

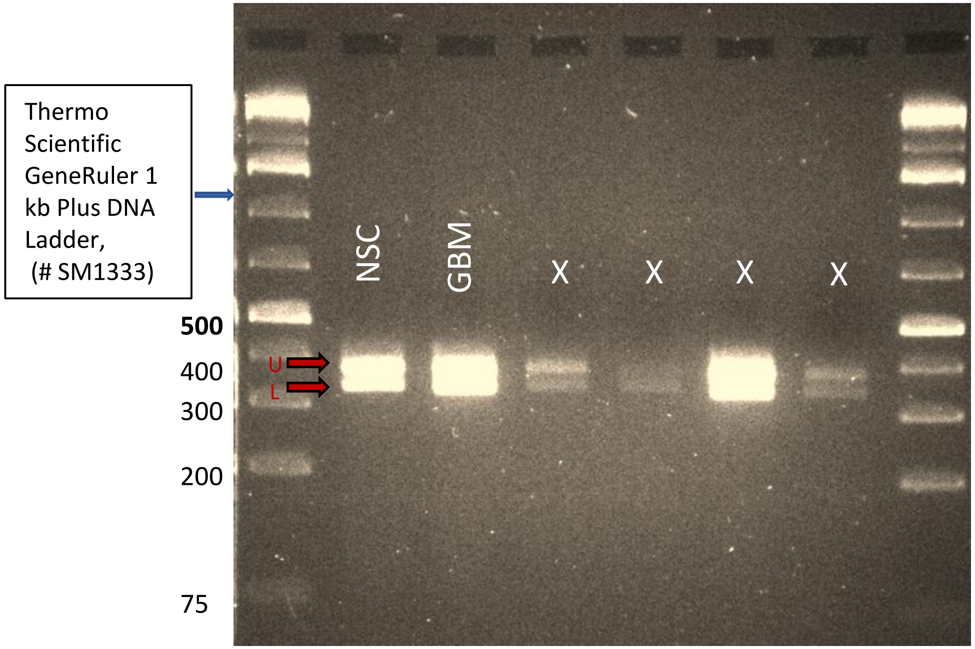

Fig 2. RFLP analysis of NANOGP8 transcript. cDNAs from NSC and GBM were amplified with NANOGP8 primers and digested with RE AlwNI. (**A**) Initial amplification yielding two bands of PCR products, pointed by red arrows are labeled as “U” and “L” for upper and lower bands respectively.
