# Supplementary material for: Analysis of regulatory sequences in exosomal DNA of NANOGP8
Source: PLoS One. 2023 Jan 25;18(1):e0280959. doi: 10.1371/journal.pone.0280959 (PMC9876286; doi:10.1371/journal.pone.0280959)
Supplement: S3 Raw image — (PDF) [file pone.0280959.s010.pdf]

Raw image for Fig 2-C

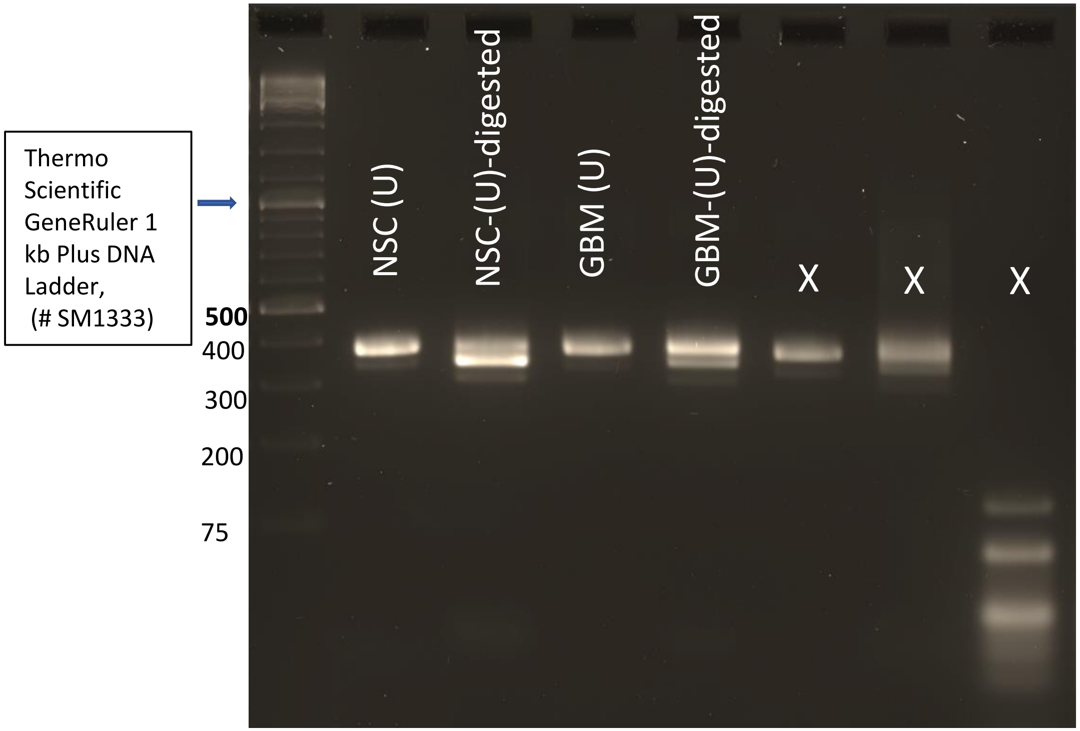

Fig 2. RFLP analysis of NANOGP8 transcript. cDNAs from NSC and GBM were amplified with NANOGP8 primers and digested with RE AlwNI. (**C**) RE-AlwNI digestion of upper bands showing partial digestion, confirming the presence of a mixed population of NANOG and NANOGP8 transcripts.
