# Supplementary material for: Analysis of regulatory sequences in exosomal DNA of NANOGP8
Source: PLoS One. 2023 Jan 25;18(1):e0280959. doi: 10.1371/journal.pone.0280959 (PMC9876286; doi:10.1371/journal.pone.0280959)
Supplement: S4 Raw image — (PDF) [file pone.0280959.s011.pdf]

Raw image for Fig 2-D

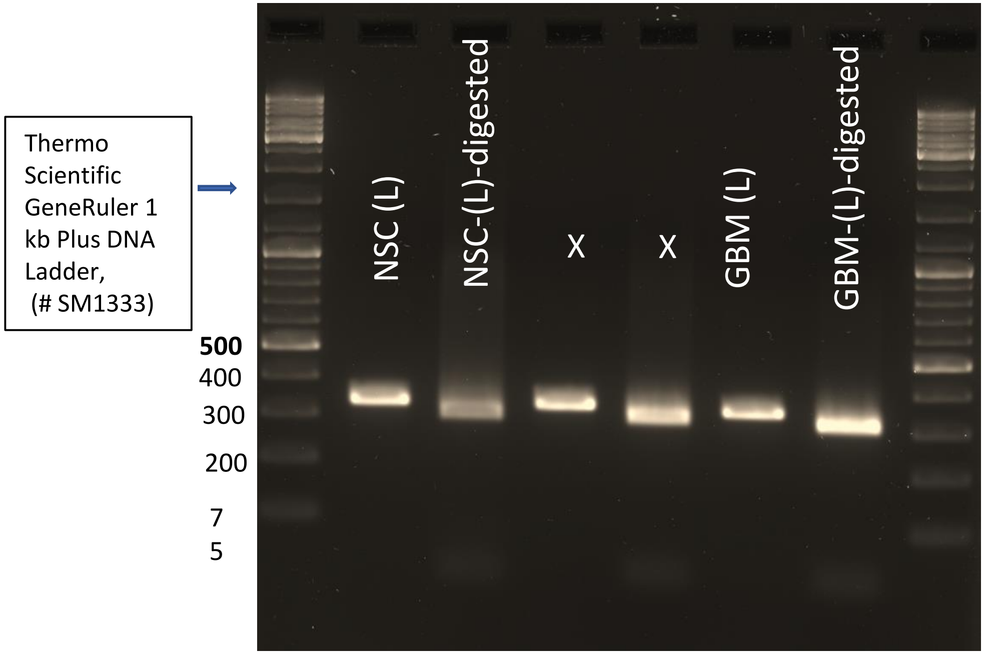

Fig 2. RFLP analysis of NANOGP8 transcript. cDNAs from NSC and GBM were amplified with NANOGP8 primers and digested with RE AlwNI. **(D)** RE-AlwNI digestion of lower bands showing complete digestion of the PCR product validates that the PCR product belongs to the NANOGP8 transcript alone. The bands for the lanes marked with “X” belong to another research project and removed in Fig 2 (D) panel.
