# Supplementary material for: Analysis of regulatory sequences in exosomal DNA of NANOGP8
Source: PLoS One. 2023 Jan 25;18(1):e0280959. doi: 10.1371/journal.pone.0280959 (PMC9876286; doi:10.1371/journal.pone.0280959)
Supplement: S5 Raw image — (PDF) [file pone.0280959.s012.pdf]

Raw image for Fig 4

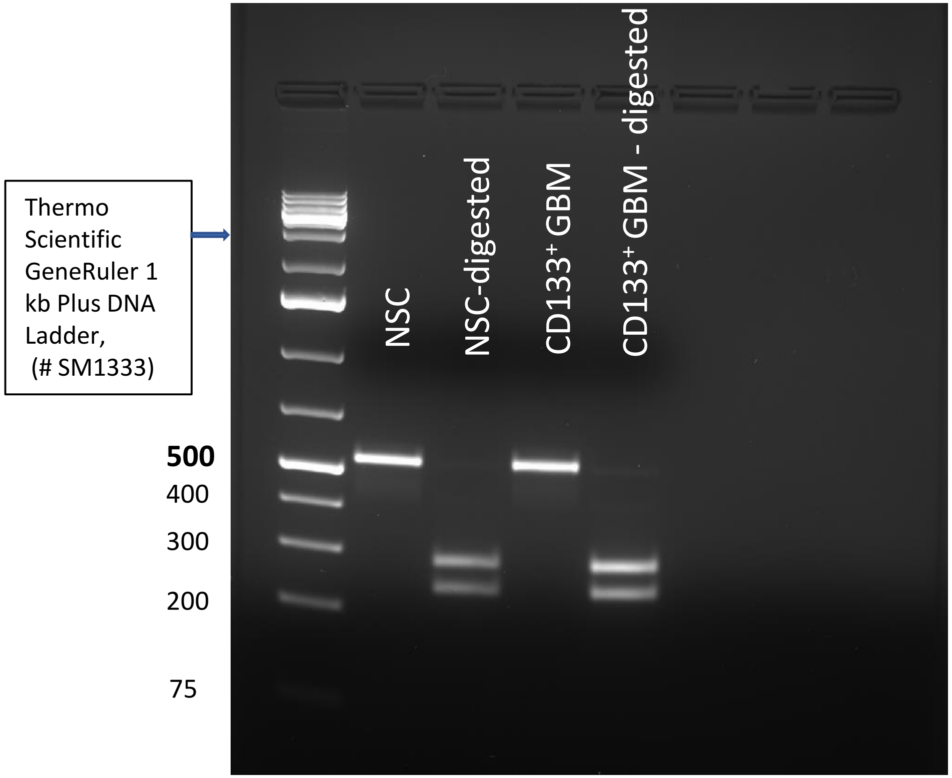

Fig 4. RFLP analysis of NANOGP8 gDNA PCR product. gDNA amplified with NANOGP8 primers. PCR product digested with HpyF3I. NSC and GBM show complete digestion, affirming the presence of the NANOGP8 transcript. A PCR product of 529 bp is digested to yield fragments of 287, 234, and 8 bp each. The 8 bp fragment is not visible in the image.
